# Tumor‐immune landscape patterns before and after chemoradiation in resectable esophageal adenocarcinomas

**DOI:** 10.1002/path.5832

**Published:** 2021-12-10

**Authors:** Tanya TD Soeratram, Aafke Creemers, Sybren L Meijer, Onno J de Boer, Wim Vos, Gerrit KJ Hooijer, Mark I van Berge Henegouwen, Maarten CCM Hulshof, Jacques JGHM Bergman, Ming Lei, Maarten F Bijlsma, Bauke Ylstra, Nicole CT van Grieken, Hanneke WM van Laarhoven

**Affiliations:** ^1^ Department of Pathology Amsterdam UMC, VU University, Cancer Center Amsterdam Amsterdam The Netherlands; ^2^ Laboratory of Experimental Oncology and Radiobiology Amsterdam UMC, University of Amsterdam, Cancer Center Amsterdam Amsterdam The Netherlands; ^3^ Department of Medical Oncology Amsterdam UMC, University of Amsterdam, Cancer Center Amsterdam Amsterdam The Netherlands; ^4^ Department of Pathology Amsterdam UMC, University of Amsterdam, Cancer Center Amsterdam Amsterdam The Netherlands; ^5^ Department of Surgery Amsterdam UMC, University of Amsterdam, Cancer Center Amsterdam Amsterdam The Netherlands; ^6^ Department of Radiotherapy Amsterdam UMC, University of Amsterdam, Cancer Center Amsterdam Amsterdam The Netherlands; ^7^ Department of Gastroenterology Amsterdam UMC, University of Amsterdam, Cancer Center Amsterdam Amsterdam The Netherlands; ^8^ Bristol‐Myers Squibb Princeton NJ USA

**Keywords:** esophageal adenocarcinoma, tumor‐immune microenvironment, PD‐L1, tumor‐infiltrating lymphocytes, chemoradiotherapy, treatment response, digital image analysis, immunohistochemistry, biomarker

## Abstract

Immunotherapy is a new anti‐cancer treatment option, showing promising results in clinical trials. To investigate potential immune biomarkers in esophageal adenocarcinoma (EAC), we explored immune landscape patterns in the tumor microenvironment before and after neoadjuvant chemoradiation (nCRT). Sections from matched pretreatment biopsies and post‐nCRT resection specimens (*n* = 188) were stained for (1) programmed death‐ligand 1 (PD‐L1, CD274); (2) programmed cell death protein 1 (PD‐1, CD279), forkhead box P3 (FOXP3), CD8, pan‐cytokeratin multiplex; and (3) an MHC class I, II duplex. The densities of tumor‐associated immune cells (TAICs) were calculated using digital image analyses and correlated to histopathological nCRT response [tumor regression grade (TRG)], survival, and post‐nCRT immune patterns. PD‐L1 positivity defined by a combined positive score of >1 was associated with a better response post‐nCRT (TRG 1–3 versus 4, 5, *p =* 0.010). In addition, high combined mean densities of CD8^+^, FOXP3^+^, and PD‐1^+^ TAICs in the tumor epithelium and stroma of biopsies were associated with a better response (TRG 1–3 versus 4, 5, *p =* 0.025 and *p =* 0.044, respectively). Heterogeneous TAIC density patterns were observed post‐nCRT, with significantly higher CD8^+^ and PD‐1^+^ TAIC mean densities compared with biopsies (both *p =* 0.000). Three immune landscape patterns were defined post‐nCRT: ‘inflamed’, ‘invasive margin’, and ‘desert’, of which ‘inflamed’ was the most frequent (57%). Compared with matched biopsies, resection specimens with ‘inflamed’ tumors showed a significantly higher increase in CD8^+^ density compared with non‐inflamed tumors post‐nCRT (*p =* 0.000). In this cohort of EAC patients, higher TAIC densities in pretreatment biopsies were associated with response to nCRT. This warrants future research into the potential of the tumor‐immune landscape for patient stratification and novel (immune) therapeutic strategies. © 2021 The Authors. *The Journal of Pathology* published by John Wiley & Sons, Ltd on behalf of The Pathological Society of Great Britain and Ireland.

## Introduction

Multimodality treatment strategies have improved outcomes of resectable esophageal cancer (EC), yet the prognosis remains disappointing [1, 2, 3]. In The Netherlands, the standard neoadjuvant treatment regimen is based on the Dutch CROSS trial [4, 5]. Although this has significantly improved survival, 34.7% of the patients have recurrent disease after a minimum follow‐up of 2 years [6].

Prognostic biomarkers harbor information on outcomes such as overall survival (OS), independent of the treatment received [7]. These biomarkers could improve survival outcomes by better patient stratification according to tumor biology, and provide clues for the development of new therapeutic strategies [8]. A systematic review with meta‐analyses in resectable esophageal adenocarcinoma (EAC) has identified biomarkers of the ‘hallmarks of cancer’ category ‘immune’ as the most significantly associated with inferior OS, compared with other categories [9]. Within this category, programmed death‐ligand 1 (PD‐L1), a ligand of the PD‐1 immune co‐inhibitory receptor, was the most prominently associated with worse OS. In addition, favorable treatment outcomes have been reported in patients with a high abundance of tumor‐infiltrating lymphocytes in resectable EAC and esophageal squamous cell carcinoma (ESC) [10, 11]. These data are particularly interesting in view of the emergent use of immunotherapies [12], especially in the setting of resectable esophageal carcinoma (EC) [13].

To date, limited data are available on the response of the tumor‐immune microenvironment (TME) to neoadjuvant chemoradiation (nCRT) in EC, and EAC in particular. The aim of this study was to assess the tumor‐immune architecture, with the objective of elucidating if these immune biomarkers are of value to predict nCRT outcomes in resectable EC. Also, as the spatial distribution of tumor‐associated immune cells (TAICs) in relation to tumor cells has been shown to influence outcomes in other tumor types [14, 15, 16, 17], we explored the spatial distribution of immune cell localization as an immune biomarker for outcome, taking a complete tumor cross‐section approach to quantify the immune landscape.

## Materials and methods

### Study cohort

The prospective surgical database of the Amsterdam University Medical Center, location AMC, was used for these retrospective analyses. Records of patients who underwent an esophagectomy between 2004 and May 2013 with histologically proven EC were identified, as previously described [18]. Records of patients with cancer of the esophagus or gastroesophageal junction (GEJ), defined as Siewert types I and II, were included and clinicopathological parameters were extracted from medical records. Subsequently, we selected only those patient records for which both a pretreatment biopsy of the primary tumor site and a matched resection specimen were available. Histopathological response was assessed by tumor regression grade (TRG) according to the Mandard score [19]. All pathological parameters, including the TRG and histological subtype, were re‐evaluated by a pathologist. Patients were treated with nCRT followed by resection, according to the CROSS regimen [4].

None of the patients received immune checkpoint inhibition. Patients receiving panitumumab treatment (*n* = 10) in addition to standard chemoradiation in the context of a phase II clinical trial were not excluded from the study because addition of panitumumab did not result in an improved treatment response or survival [20]. The formalin‐fixed, paraffin‐embedded (FFPE) material was retrieved in compliance with the revised Declaration of Helsinki, 2004 [21].

### Immunohistochemistry

**Figure 1:**
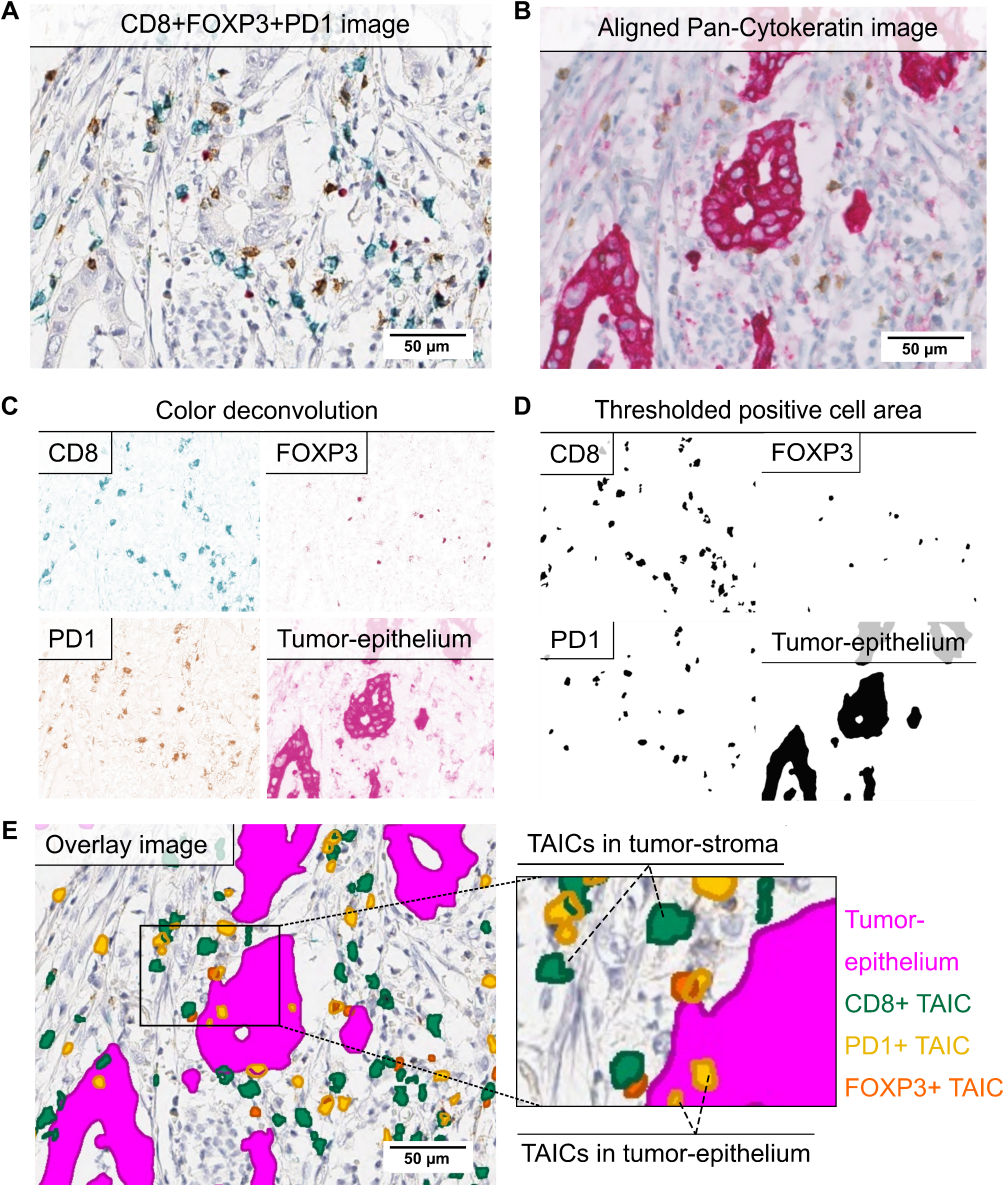
Multiplex immunohistochemistry (mIHC) of CD8, FOXP3, PD‐1, and pan‐cytokeratin. (A) Image of triplex CD8 + FOXP3 + PD‐1 IHC assay. CD8^+^ cells were visualized with Vina Green (blue/green color), FOXP3^+^ cells with Vulcan Red (red color), and PD‐1^+^ cells in DAB (brown color). (B) Image of mIHC assay after the stripping step and staining of tumor/epithelial cells with pan‐cytokeratin in Fast Red (red color). PD‐1^+^ cells were still visible in brown. (C) Color‐separated images of CD8^+^, FOXP3^+^, PD‐1^+^ and pan‐cytokeratin^+^ cells after digital color deconvolution of all chromogens. (D) Binary images of positive detected CD8^+^, FOXP3^+^, PD‐1^+^, and tumor cells after application of thresholds and filters. (E) Overlay image with annotated positive cells on original triplex IHC image. CD8^+^ cells are annotated in green, FOXP3^+^ cells in orange, PD‐1^+^ cells in yellow, and tumor epithelium in magenta. TAICs were classified as TAICs located in the tumor stroma or tumor epithelium. All images were taken at 20× objective magnification. Scale bars: 50 μm. TAIC, tumor‐associated immune cell.

Selected FFPE blocks from biopsies and resection specimens were sectioned at 4 μm thickness and slides were checked for vital tumor cells via a consecutive hematoxylin and eosin (H&E)‐stained slide. H&E slides were also used to assess TAIC density patterns (supplementary material, Table S1). Matched slides of the pretreatment biopsies and resection specimens were simultaneously stained with: i, the rabbit monoclonal antibody for PD‐L1 (clone 28‐8; Abcam, Cambridge, MA, USA), visualized with DAB chromogen (Dako, Carpinteria, CA, USA); ii, a duplex with the MHC I mouse monoclonal antibody (clone EMR8‐5; Abcam), visualized with Vulcan Fast Red chromogen (Biocare Medical, Concord, CA, USA), and the MHC II mouse monoclonal antibody (clone CR3/43; Dako), visualized with StayYellow chromogen (Abcam); and iii, a multiplex stain with the Forkhead box P3 (FOXP3) mouse monoclonal antibody (clone 236A/E7; Abcam), visualized with Vulcan Fast Red chromogen; the PD‐1 rabbit monoclonal antibody [clone EPR4877(2); Abcam], visualized with DAB chromogen (Dako); and the CD8 mouse monoclonal antibody (clone C8/144B; Dako), visualized with Vina Green chromogen (Biocare Medical) (Figure 1A). All slides were scanned using the Aperio ScanScope AT Turbo system (Aperio, Vista, CA, USA). After a stripping step of CD8 + FOXP3 + PD‐1 triplex slides, the epithelial/tumor cells were stained in the Ventana Benchmark Ultra Slide Stainer (Roche Tissue Diagnostics, Roche, Basel, Switzerland) with a pan‐cytokeratin primary antibody (clone BS5; dilution 1:25; Nordic BioSite, Täby, Sweden) (iv) and visualized using Ultraview Alkaline Phosphatase Red (Roche) (Figure 1B). All anti‐pan‐cytokeratin‐stained slides were subsequently digitized on the Philips IntelliSite Ultra‐Fast Scanner (Philips Digital Pathology Solutions, Best, The Netherlands). The CD8 + FOXP3 + PD‐1 multiplex (iii) was not assessed in cases with no remaining tumor cells in post‐treatment resection specimens (TRG 1).

The MHC I + MHC II duplex and CD8 + FOXP3 + PD‐1 triplex immunohistochemical (IHC) assays were performed and validated at the CAP/CLIA accredited Mosaic Laboratories (Lake Forest, CA, USA) in accordance with Mosaic Laboratories' standard operating procedures (see Supplementary materials and methods for details on methods and validation).

### Image analyses

#### Programmed death‐ligand 1 (PD‐L1)

PD‐L1 expression and intensity on tumor cells were scored as a percentage of total tumor cells. PD‐L1 expression on TAICs was categorized according to the percentage of positive cells (Supplementary materials and methods and supplementary material, Table S2). The combined positive score (CPS) was calculated by dividing the number of PD‐L1^+^ tumor cells and PD‐L1^+^ immune cells by the total number of tumor cells present in specimens with at least 100 vital tumor cells.

#### MHC I + II duplex

The MHC class I and II duplexes (ii) were scored by digital image analyses using the Cytonuclear IHC module from HALO image analysis software (Indica Labs, Albuquerque, NM, USA) and visual quantification by a trained pathologist. MHC I expression and MHC II expression were defined as the percentage of positive tumor cells and the percentage of positive stromal cells, respectively. Details are provided in Supplementary materials and methods and supplementary material, Table S3.

#### CD8 + FOXP3 + PD‐1 + pan‐cytokeratin multiplex

Combined whole‐mount CD8 + FOXP3 + PD‐1 + pan‐cytokeratin (iii + iv)‐stained slides were analyzed with open‐source software QuPath version 0.1.2 (Queen's University, Belfast, UK) [22] and ImageJ (NIH, Bethesda, MD, USA) [23]. Each image was divided into a grid of tiles of 2 mm^2^ in QuPath. Tumor‐containing tiles were manually classified as ‘tumor center’ (TC) or ‘invasive margin’ (IM) tiles. Tiles were further processed in ImageJ in an automated manner. Steps included alignment of CD8 + FOXP3 + PD‐1 and pan‐cytokeratin images, tissue segmentation, and positive cell detection using ImageJ autothreshold (Figure 1C,D). Positive cells were detected separately in segmented tumor epithelium (pan‐cytokeratin^+^ area) and tumor stroma (negative for pan‐cytokeratin, but within the same tumor‐containing tile) (Figure 1E). Tiles without pan‐cytokeratin^+^ tumor cells were classified as non‐tumor stroma. Tumor epithelium and tumor stroma combined was defined as the tumor compartment. Single and double positive cells were identified in the entire cross section of the tumor, but only the single positive TAICs were included in further analyses. The number, location, density (cells/mm^2^ tissue) of each cell type, and the ratio of TAICs between TC and IM was calculated in RStudio (RStudio, PBC, Boston, MA, USA) (for details see Supplementary materials and methods).

#### Immune landscape classification

Tumors were categorized into the immune landscapes ‘inflamed’, ‘invasive margin’, and ‘desert’ based on the mean TAIC density of the tiles in TC and IM, as well as the ratio of TC/IM (supplementary material, Table S4). Tumors with a high TAIC density (≥75) in TC were classified as ‘inflamed’. Tumors with a high density in IM (≥400), a low density in TC (<75), and with a TC/IM ratio less than 0.5 were classified as ‘invasive margin’. Tumors with a low TAIC density in both compartments were classified as ‘desert’.

### Statistical analysis

Differences in biomarker expression and clinicopathological variables were assessed using Pearson's chi‐squared test or Fisher's exact test. For unpaired analyses without a normal distribution, the non‐parametric Wilcoxon rank test and Kruskal–Wallis test were used to compare ranks. Univariate and multivariate logistic regression analyses were performed to check for associations between biomarker expression and histopathological treatment response, including variables with a known association with the dependent variable. Survival analyses were performed using Kaplan–Meier and multivariable Cox proportional hazard regression analysis, including variables with a reported association with prognosis [24]. OS was computed from the date of diagnosis to the date of death and censored for a non‐cancer‐related cause of death; surviving patients were censored at the date of last follow‐up. Statistical analyses were performed in IBM SPSS statistics 24.0 (IBM, Armonk, NY, USA) and RStudio. *p* < 0.05 was regarded as statistically significant.

## Results

### Immune landscape and PD‐L1 expression in pretreatment biopsies

**Figure 2:**
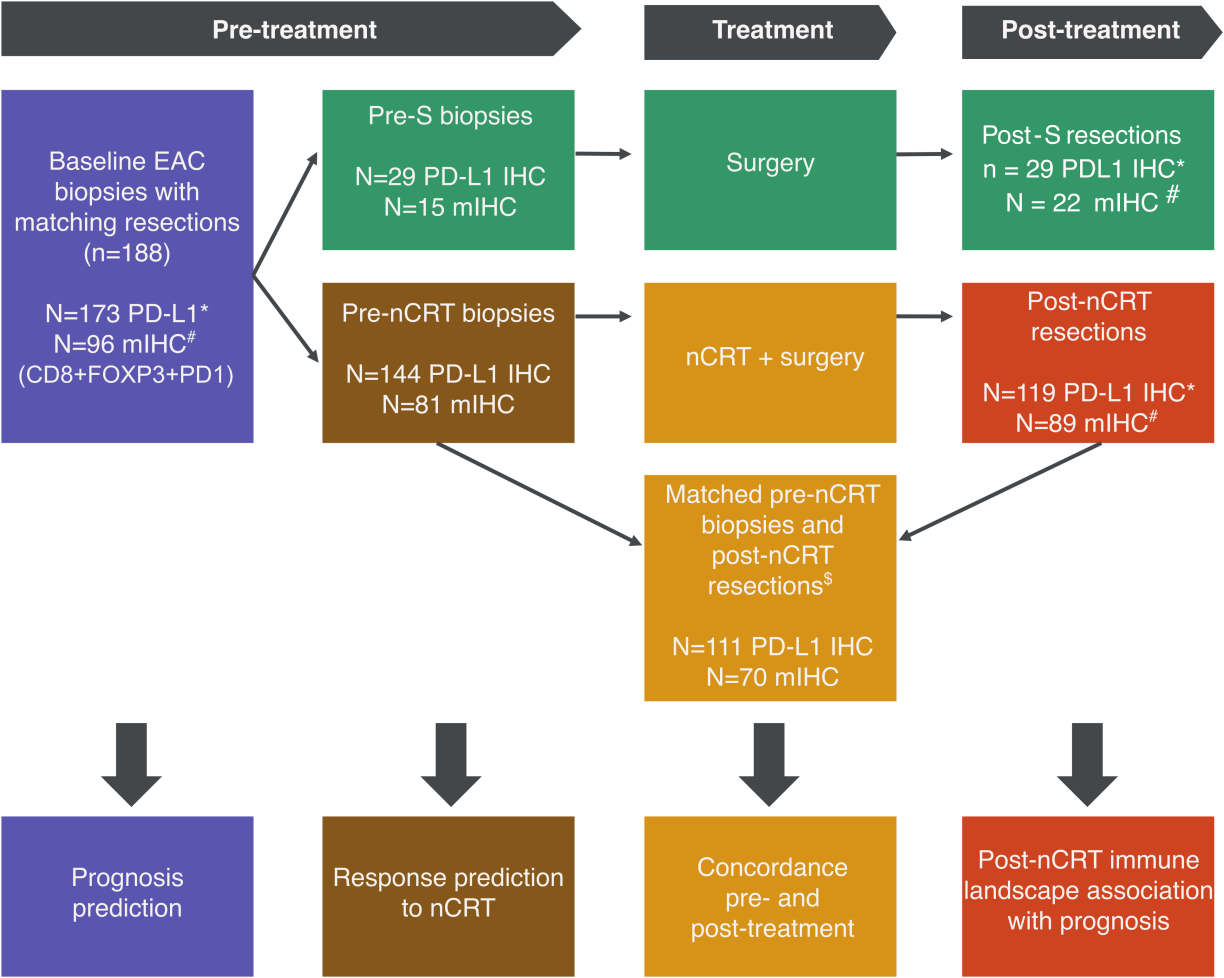
Flowchart of the patients included and the assays and analyses performed. Patients were excluded from analysis if: *PD‐L1 IHC was not evaluable due to too few or no viable tumor cells left in tissue; ^#^multiplex IHC (IHC of CD8, FOXP3, PD‐1, and pan‐cytokeratin) was not evaluable due to failed pan‐cytokeratin IHC, failed cell detection in image analysis, no tumor tissue left, or failed digital scan; ^$^no matching biopsy or resection was available for analysis. nCRT, neoadjuvant chemoradiation; S, surgery.

To characterize the immune landscape in EAC before and after nCRT, matched pretreatment biopsies and post‐treatment resections of 188 patients were analyzed by single and multiplex IHC. Multiplex IHC of cytotoxic (CD8^+^), regulatory (FOXP3^+^), and immune checkpoint positive (PD‐1^+^) TAICs was performed in 96 biopsies and 89 resections, of which 70 were matched. PD‐L1 IHC was performed in 173 biopsies and 119 resections, of which 111 were matched. Several samples were excluded due to technical failure of the methods (flowchart in Figure 2).

**Figure 3:**
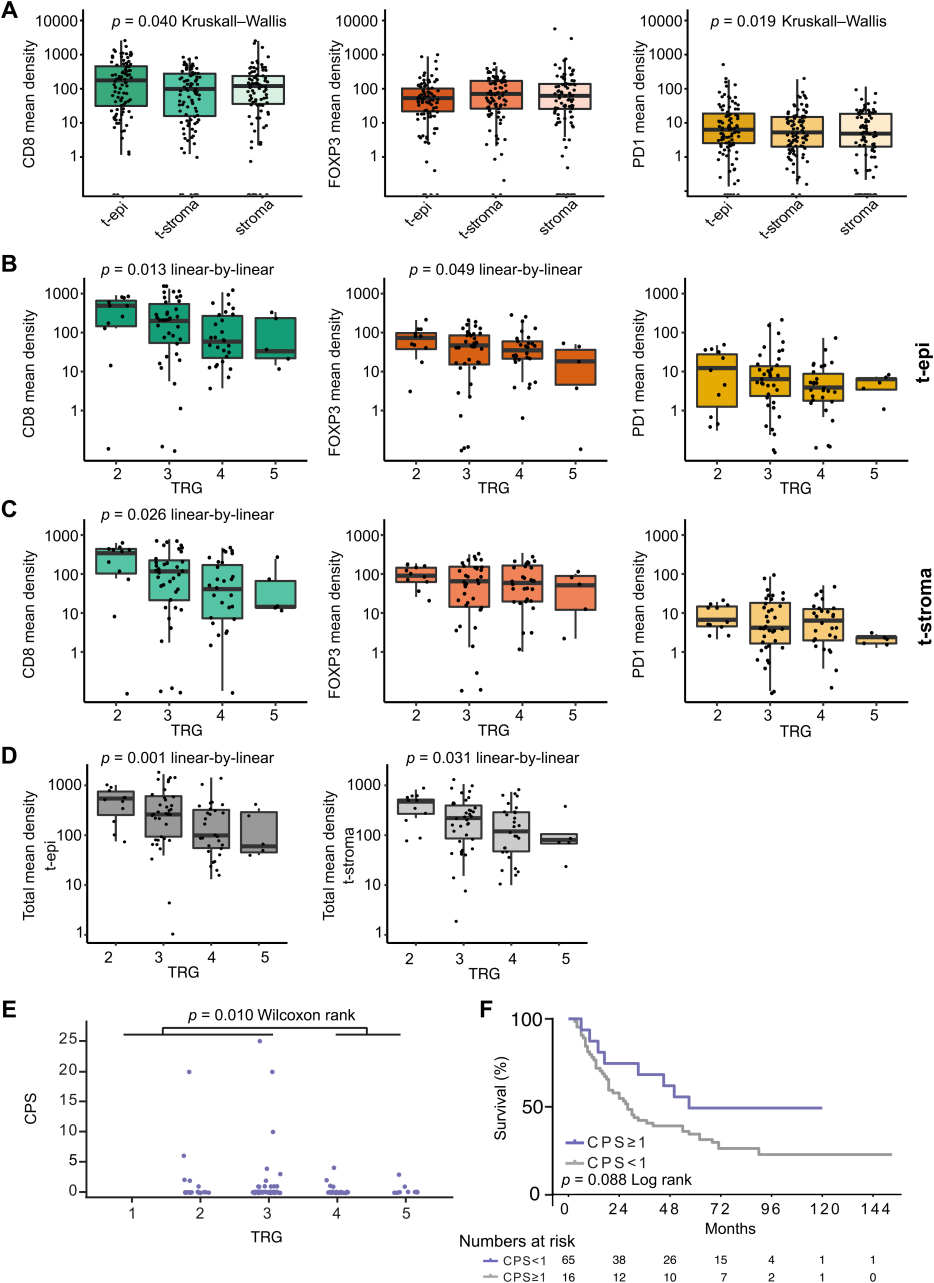
Tumor‐associated immune cells (TAICs) in pretreatment EAC biopsies. (A) The mean densities (cells/mm^2^) of CD8^+^, FOXP3^+^, and PD‐1^+^ TAICs in the tumor epithelium (t‐epi), tumor stroma (t‐stroma), and non‐tumor stroma (stroma) of pretreatment EAC biopsies. The Kruskal–Wallis test was used to detect overall difference of TAIC density between the three compartments. (B) The mean densities (cells/mm^2^) of CD8^+^, FOXP3^+^, and PD‐1^+^ TAICS in the tumor epithelium per tumor regression grade (TRG) of pretreatment EAC biopsies. (C) The mean densities (cells/mm^2^) of CD8^+^, FOXP3^+^, and PD‐1^+^ TAICs in the tumor stroma per TRG of pretreatment EAC biopsies. (D) The combined mean densities (cells/mm^2^) of CD8^+^, FOXP3^+^, and PD‐1^+^ TAICs in the tumor stroma per TRG of pretreatment EAC biopsies. (E) PD‐L1 expression by CPS in pretreatment biopsies versus TRG. The Wilcoxon rank sum test was used to detect differences in CPS between TRG low (1–3) and TRG high (4, 5) scores. (F) Kaplan–Meier analyses of the overall survival (OS) difference between PD‐L1‐negative (CPS < 1) and PD‐L1‐positive (CPS ≥ 1) pre‐nCRT biopsies. The log‐rank test was used to detect significant survival differences. (A–D) Linear‐by‐linear chi squared test was used to detect significant linear association of TAIC density and ordinal TRG scores. TAIC density in log_10_ scale (*Y*‐axis).

TAICs were present in the stroma surrounding the tumor cells (tumor stroma) or were in close contact with tumor cells (tumor epithelium). TAICs in the tumor epithelium are more likely to affect tumor cells due to local cytokine effects and direct cell interaction. We therefore assessed the TAICs in both the tumor epithelium and the tumor stroma, as well as in the adjacent non‐tumor stroma. The tissue size and percentage of biopsied tumor cells varied per biopsy (supplementary material, Figure s1A). To correct for this, densities were computed for the CD8^+^, FOXP3^+^, and PD‐1^+^ TAICs in cells/mm^2^ (supplementary material, Table S5). Statistically significant differences were seen between the mean number of CD8^+^ and PD‐1^+^ TAICs in the different compartments, with higher numbers in tumor epithelium (Figure 3A, *p =* 0.040 and *p =* 0.019, Kruskal–Wallis test, respectively).

PD‐L1 is suggested to be preferentially expressed on immune cells in EAC [25]. Therefore, PD‐L1 expression was determined on tumor cells as well as on TAICs. Tumor expression of PD‐L1 was detected in a small subset of the patients (9.2%, *n* = 16), was of low intensity, and was present in a small percentage of tumor cells (supplementary material, Figure S2A). PD‐L1^+^ TAICs were detected in 23.1% (*n* = 40) of the pretreatment biopsies (supplementary material, Figure S2B), of which 19.6% (*n* = 34) had a low mean density (1–4%). PD‐L1^+^ TAICs were mostly located in the tumor epithelium (supplementary material, Figure S2B).

The interaction between the different markers was explored by a correlation matrix. CD8^+^, FOXP3^+^, and PD‐1^+^ TAIC densities were positively correlated in both the tumor epithelium and the tumor stroma compartment (supplementary material, Figure S1B), suggesting a general T‐cell infiltration. There was no correlation between TAIC density and MHC I or MHC II expression, or with PD‐L1 expression (CPS).

### TAIC density in pretreatment biopsies is associated with histopathological response

Next, the association of CD8^+^, FOXP3^+^ or PD‐1^+^ TAICs with TRG was examined to determine whether this could serve as a biomarker for response to nCRT (supplementary material, Table S6). Pretreatment biopsies of patients with lower TRG scores post‐nCRT had higher cell densities of CD8^+^ and FOXP3^+^ TAICs in the tumor epithelium compartment (Figure 3B, *p* = 0.013 and *p =* 0.049, linear‐by‐linear trend test, and supplementary material, Figure S3). In the tumor stroma compartment, only higher cell densities of CD8 showed a significantly improved response to nCRT (Figure 3C, *p* = 0.026, linear‐by‐linear trend test); however, a similar trend was seen for high FOXP3^+^ and PD‐1^+^ TAIC density in low TRG (Figure 3C). Since CD8^+^, PD‐1^+^, and FOXP3^+^ TAICs were correlated, the combined mean density of CD8^+^, FOXP3^+^, and PD‐1^+^ TAICs was calculated for the tumor epithelium and tumor stroma compartments, as a surrogate marker for general T‐cell infiltration. A higher combined mean density in both compartments was associated with a better pathological response (Figure 3D, *p =* 0.001 and *p =* 0.031, linear‐by‐linear trend test). These data suggest that TAICs in the tumor stroma also play a role in the response to nCRT, even though they are not in direct contact with tumor cells.

PD‐L1 expression determined by CPS combines tumor and immune cell expression and has been suggested as a biomarker for immunotherapy response [26]. Therefore, CPS was calculated to determine the association with response to nCRT. CPS > 1 (*n* = 16) was associated with lower TRG (TRG 1–3) (Figure 3E, *p* = 0.010, Wilcoxon rank).

In univariate analysis, CPS and combined mean density were significant predictors for response (TRG 1–3 versus 4, 5; Table 1). Improved treatment response was mainly associated with the mean density of CD8^+^ TAICs in the tumor epithelium and tumor stroma compartments (supplementary material, Table S7). Multivariate logistic regression analysis was performed to control for clinical parameters and included CPS > 1 as well as combined mean density. In this analysis, none of the variables remained significant predictors (Table 1).

**Table 1:** Uni‐ and multi‐variate regression to predict TRG 1–3 versus TRG 4, 5 in pre‐nCRT biopsies.

	Fisher's exact test			Univariate logistic regression				Multivariate logistic regression			
	Mandard low	Mandard high	*P* value	OR	95% CI lower	95% CI upper	*P* value	OR	95% CI lower	95% CI upper	*P* value
	*n* = 48	*n* = 33									
**Age**			0.498	0.967	0.919	1.019	0.209	1.048	0.984	1.116	0.149
<60	21 (65.6%)	11 (34.4%)									
>60	27 (55.1%)	22 (44.9%)									
**T‐stage**			0.489	0.982	0.370	2.604	0.971				0.88
1	0 (0.0%)	1 (100%)						5.5E+08	0	NA	1
2	8 (72.7%)	3 (27.3%)						0.372	0.018	7.638	0.521
3	38 (57.6%)	28 (42.4%)						0.67	0.046	9.83	0.77
4	2 (66.7%)	1 (33.3%)									
**N‐stage**			0.980	0.945	0.525	1.700	0.850				0.573
0	11 (61.1%)	7 (38.9%)						0.381	0.043	3.393	0.387
1	33 (58.9%)	23 (41.1%)						0.333	0.043	2.566	0.291
3	4 (57.1%)	3 (42.9%)									
**CPS ≥ 1**			**0.010**	0.157	0.033	0.745	**0.020**	5.184	0.927	28.995	0.061
No	34 (52.3%)	31 (44.7%)									
Yes	14 (87.5%)	2 (12.5%)									
**CPS ≥ 10**			0.142	0.000	0.000	NA	0.999				
No	44 (57.1%)	33 (42.9%)									
Yes	4 (100%)	0 (0%)									
**Total mean density tumor epithelium**				1.002	1.000	1.003	**0.025**	0.998	0.995	1.002	0.318
**Total mean density tumor stroma**				1.002	1.000	1.004	**0.044**	0.999	0.995	1.004	0.798

*P* values in bold are statistically significant.

CPS, combined positive score; NA, not available.

### Correlation of pretreatment TAICs with OS

Since CPS and TAIC density were associated with response to nCRT, the potential survival benefit of patients with high expression of these biomarkers was investigated. No significant survival difference between CPS‐high (≥1) and CPS‐low (<1) patients was found (Figure 3F, *p* = 0.088, log rank; HR 1.889; CI 0.892–3.999). When assigning patients into low and high groups using the median of CD8, PD‐1, and FOXP3 density of the cohort as a cut point, patients with high PD‐1^+^ TAIC densities had a significantly worse OS compared with patients with low densities (*n* = 41 versus *n* = 40, median OS 46 months versus 30 months, *p =* 0.045, log rank). Other TAIC markers were not associated with survival outcome (data not shown).

To correct for other prognostic markers, a multivariate analysis was performed. The mean density of CD8^+^, in the tumor epithelium and tumor stroma compartments, was associated with OS (HR 1.003, CI 1.00–1.005, *p =* 0.020 and HR 0.996, CI 0.992–1.000, *p =* 0.039, respectively; supplementary material, Table S8), as well as with the combined mean density (*p* = 0.004 and *p* = 0.009, Table 2). Since in univariate analysis these variables were not significant, these data suggest that TAIC density is only prognostic in a subgroup of patients, when other prognostic factors are taken into account.

**Table 2:** Uni‐ and multi‐variate Cox regression to predict OS in pre‐nCRT biopsies.

	Fisher's exact test			Univariate Cox regression				Multivariate Cox regression			
	Alive	Deceased	*P* value	HR	95% CI lower	95% CI upper	*P* value	HR	95% CI lower	95% CI upper	*P* value
	*n* = 25	*n* = 56									
**Age**			0.156	1.013	0.982	1.045	0.410	1.018	0.985	1.052	0.290
<60	3 (15.8%)	15 (84.2%)									
>60	22 (35.5%)	40 (64.5%)									
**T‐stage**			0.090				0.356				0.550
1	0 (0.0%)	1 (100%)		3.260	0.202	52.570	0.405	2.881	0.158	52.510	0.475
2	6 (54.5%)	5 (45.5%)		1.307	0.152	11.208	0.807	1.792	0.184	17.451	0.615
3	17 (25.8%)	49 (74.2%)		2.686	0.370	19.497	0.328	2.956	0.368	23.743	0.308
4 (ref)	2 (66.7%)	1 (33.3%)									
**N‐stage**			0.457				0.608				0.876
0	7 (38.9%)	11 (61.1%)		0.946	0.301	2.975	0.925	0.725	0.212	2.477	0.608
1	15 (26.8%)	41 (73.2%)		1.297	0.464	3.626	0.620	0.769	0.245	2.416	0.653
3 (ref)	3 (42.9%)	4 (57.1%)									
**CPS ≥ 1**			0.077	1.889	0.893	3.999	0.096	2.379	0.972	5.824	0.058
No	17 (26.2%)	48 (73.8%)									
Yes (ref)	8 (50%)	8 (50%)									
**CPS ≥ 10**			1.000	0.823	0.257	2.637	0.742				
No	24 (31.2%)	53 (68.8%)									
Yes	1 (25%)	3 (75%)									
**Total mean density tumor epithelium**				1.000	0.999	1.001	0.947	1.002	1.001	1.004	**0.004**
**Total mean density tumor stroma**				0.999	0.998	1.000	0.246	0.997	0.995	0.999	**0.009**

*P* values in bold are statistically significant.

CPS, combined positive score.

### Heterogeneous spatial TAIC distribution in post‐nCRT resection specimens

**Figure 4:**
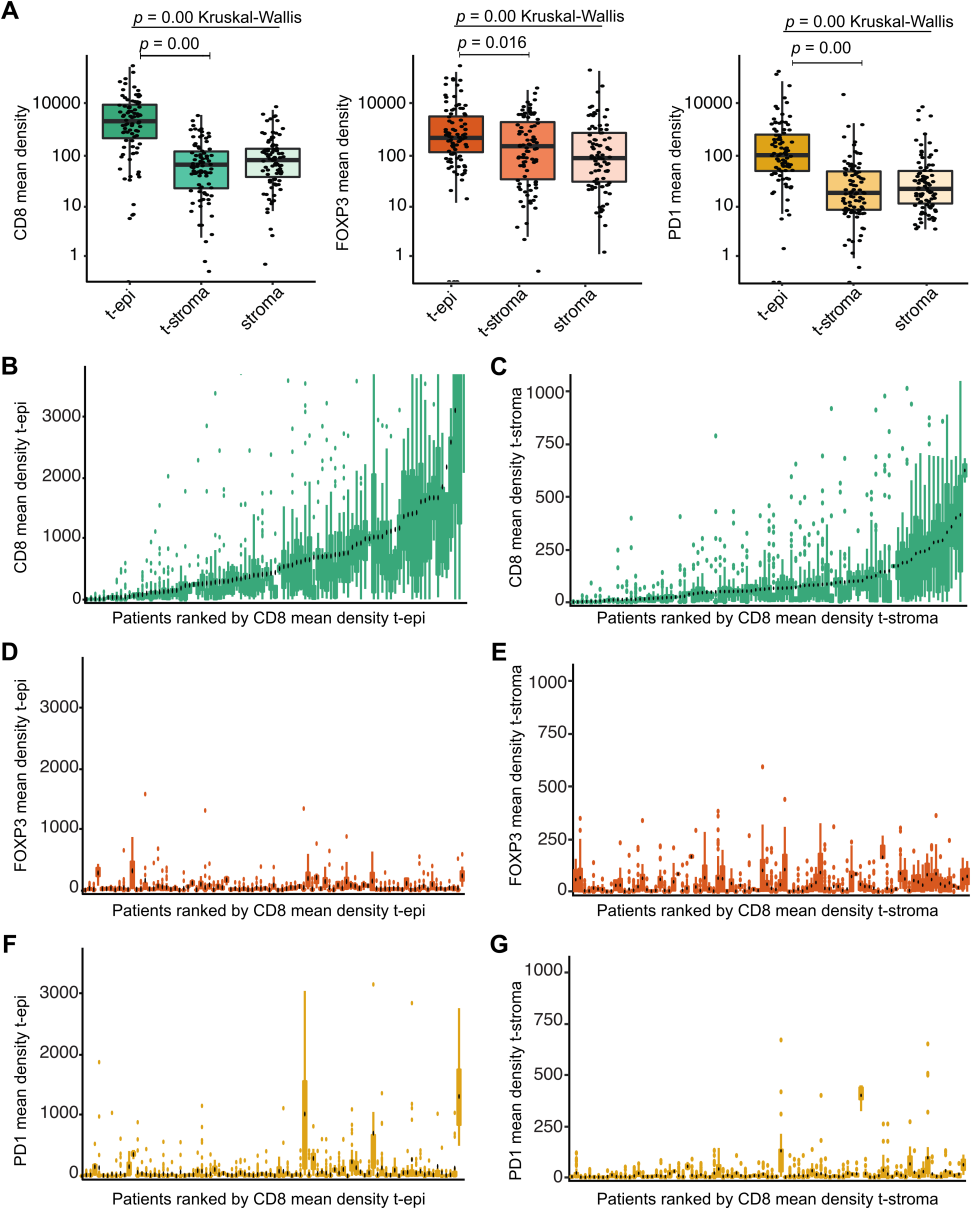
Heterogeneity in tumor‐associated immune cell (TAIC) density in EAC resections post‐nCRT. (A) The mean densities (cells/mm^2^) of CD8^+^, FOXP3^+^, and PD‐1^+^ TAICs in the tumor epithelium (t‐epi), tumor stroma (t‐stroma), and non‐tumor stroma (stroma) of post‐nCRT resection specimens. The Kruskal–Wallis test was used to detect overall difference of TAIC density between the three compartments. *Post hoc* analysis was performed with the pairwise Wilcoxon rank sum test and Benjamini–Hochberg *P* value adjustment. TAIC density in log_10_ scale (*Y*‐axis). (B, C) The mean densities (cells/mm^2^) of CD8^+^ TAICs in (B) the tumor epithelium (t‐epi) and (C) tumor stroma (t‐stroma). Patients are ranked by CD8 mean density. (D, E) The mean densities (cells/mm^2^) of FOXP3^+^ TAICs in (D) the tumor epithelium (t‐epi) and (E) tumor stroma (t‐stroma). Patients are ranked by CD8 mean density. (F, G) The mean densities (cells/mm^2^) of PD‐1^+^ TAICs in (F) the tumor epithelium (t‐epi) and (G) tumor stroma (t‐stroma). Patients are ranked by CD8 mean density.

The post‐nCRT immune landscape was analyzed in whole resection slides (supplementary material, Table S5), capturing the variation in TAIC density within one slide. The mean CD8^+^, FOXP3^+^, and PD‐1^+^ TAIC densities were significantly higher in the tumor epithelium than in the tumor stroma and non‐tumor stroma compartments (Figure 4A, *n* = 89, *p =* 0.00, *p =* 0.016, *p =* 0.00, respectively; Kruskal–Wallis with pairwise Wilcoxon rank sum comparisons). When the CD8^+^, FOXP3^+^, and PD‐1^+^ TAIC densities of all individual tiles were plotted, large intratumoral heterogeneity was observed in the tumor epithelium, tumor stroma, and non‐tumor stroma compartments (Figure 4B–G). The mean densities of CD8^+^, FOXP3^+^, and PD‐1^+^ TAICs in post‐nCRT resections did not correlate with clinicopathological outcome parameters (data not shown), possibly due to the heterogeneous spatial distribution of TAICs.

### Immune landscape patterns in post‐nCRT resections are predominantly inflamed

**Figure 5:**
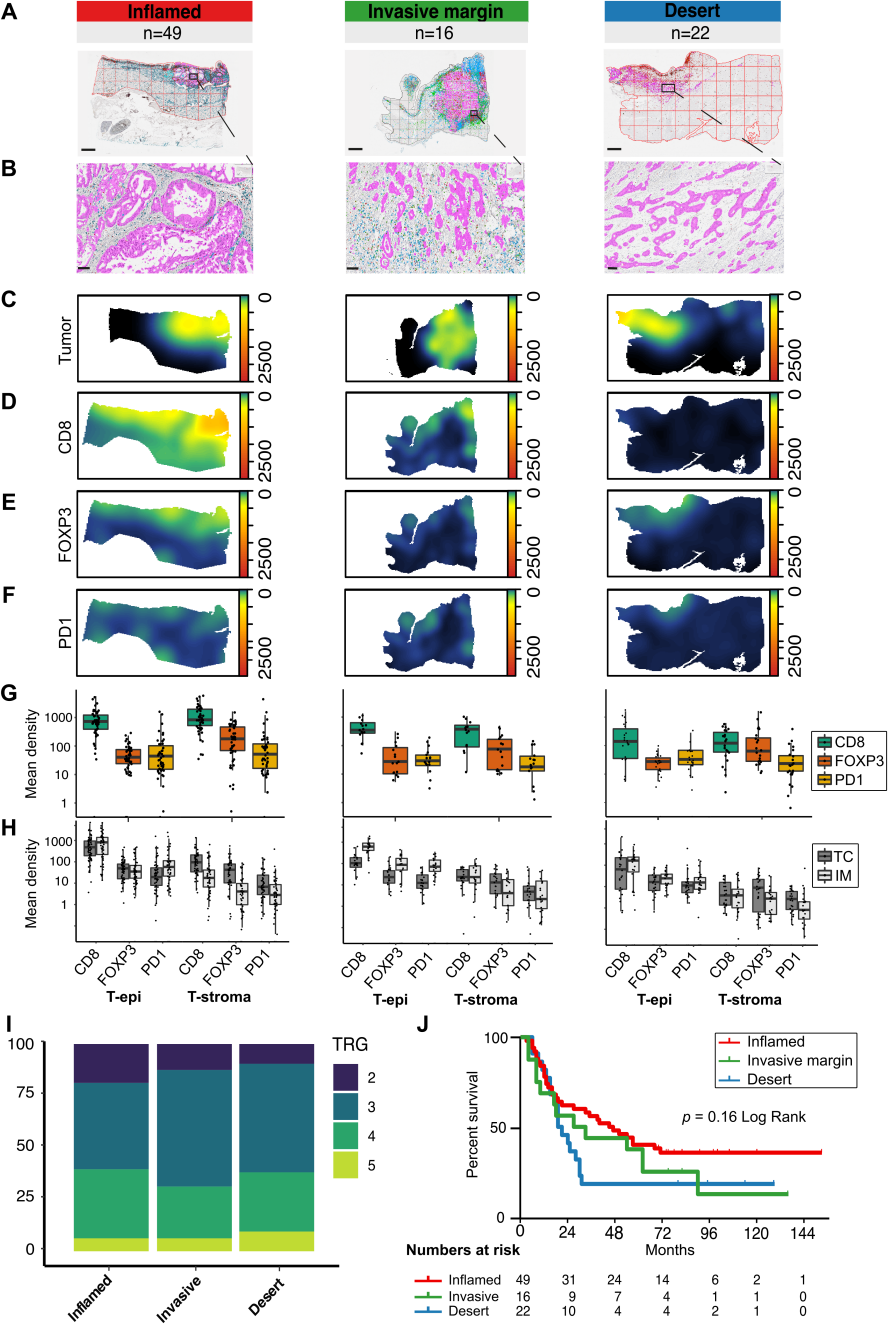
The tumor‐immune landscape in post‐nCRT EAC resection specimens. (A) Representative images of digitally annotated CD8^+^ (blue), FOPX3^+^ (red), and PD‐1^+^ (brown) TAICs, and pan‐cytokeratin‐positive tissue (magenta) per immune‐landscape pattern. Scale bar: 2 mm. (B) Zoom of the images depicted in A. Scale bar: 100 μm. (C–F) Density heat maps of (C) the tumor cell density, (D) the CD8^+^ TAIC density, (E) the FOXP3^+^ TAIC density, and (F) the PD‐1^+^ TAIC density of representative images per pattern. (G) Mean cell densities (cells/mm^2^) in the tumor epithelium (t‐epi) and tumor stroma (t‐stroma) for CD8^+^, FOXP3^+^, and PD‐1^+^ TAICs per immune‐landscape pattern. Mean TAIC density depicted in log_10_ scale (*Y*‐axis). (H) Mean cell densities (cells/mm^2^) in the tumor center (TC) and invasive front (IM) in the tumor epithelium (t‐epi) and tumor stroma (t‐stroma) for CD8^+^, FOXP3^+^, and PD‐1^+^ TAICs per immune‐landscape pattern. (I) The percentage of EAC resection specimens post‐nCRT per TRG per immune‐landscape pattern. (J) The difference in OS per immune‐landscape pattern; *p =* 0.16 in the log‐rank test of Kaplan–Meier analysis.

The role of spatial distribution of TAICs in the tumor microenvironment was further explored by categorizing the tumors into previously described immune landscape patterns, such as ‘inflamed’ (or ‘hot’), ‘invasive margin’ (or ‘excluded’), and ‘immune desert’ (or ‘cold’) [27]. To determine whether these immune subtypes were present in post‐treatment resections in EAC, cell‐specific heat maps were generated to visualize the immune landscape patterns (Figure 5A–H). All tumors could be categorized into these patterns based on the combined mean CD8^+^, FOXP3^+^, and PD‐1^+^ densities in the tumor center and invasive front, and based on the ratio of combined density in tumor center/invasive front (supplementary material, Tables S4 and S10). The majority of patients had an inflamed immune landscape pattern in post‐nCRT resections (*n* = 49 of 87, 56%).

Inflamed tumors have been suggested to exhibit an activated immune state directed against the tumor [28], but the post‐nCRT spatial distribution patterns were not associated with histopathological response (Figure 5I) or with OS (Figure 5J). In uni‐ and multi‐variate Cox regression, no significant predictors of OS were identified (supplementary material, Table S9).

### Higher CD8‐positive TAIC densities in EAC resection specimens after nCRT compared with pretreatment biopsies

**Figure 6:**
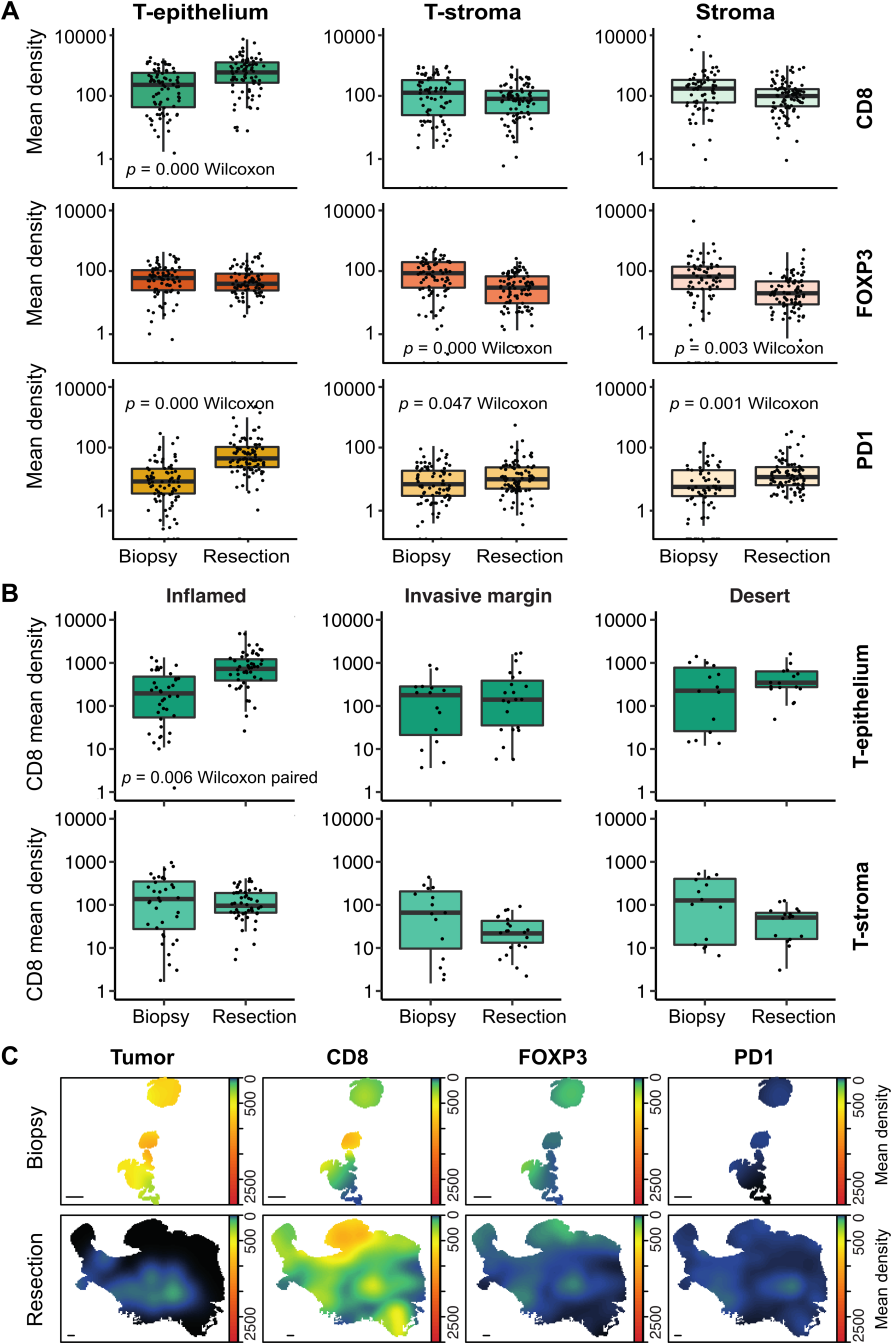
The difference in immune landscapes in pretreatment biopsies and post‐nCRT resection specimens. (A) The difference in mean CD8^+^, FOXP3^+^, and PD‐1^+^ TAIC density (cells/mm^2^) between pretreatment biopsies and post‐nCRT resection specimens in the tumor epithelium (T‐epithelium), tumor stroma (T‐stroma), and non‐tumor stroma (Stroma). (B) The difference in CD8^+^ mean density (cells/mm^2^) between pretreatment biopsies and post‐nCRT resection specimens in the tumor epithelium (T‐epithelium) and tumor stroma (T‐stroma) per immune‐landscape pattern. (C) Tumor cells and CD8^+^, FOXP3^+^, and PD‐1^+^ TAIC density heat maps of a representative biopsy with matched inflamed post‐nCRT resection specimen. Scale bar: 1 mm. (A, B) Statistical differences between biopsy and resection specimens were determined by the paired Wilcoxon signed‐rank test. The *Y*‐axis is depicted in log_10_ scale.

Considering that the inflamed immune landscape was frequently present post‐nCRT, a potential treatment effect on TAICs was examined by comparing the TAIC densities in matched biopsy and resection specimens. Compared with pretreatment biopsies, significantly higher mean densities of CD8^+^ and PD‐1^+^ TAICs were detected in the tumor epithelium post‐nCRT (both *p =* 0.000, paired Wilcoxon signed‐rank test) (Figure 6A). To assess whether this was a general increase after nCRT or specific to a subset of tumors, the pre‐ and post‐treatment densities were compared between the immune subtypes determined post‐nCRT. Compared with the non‐inflamed categories, invasive margin and desert, those patients with inflamed tumors showed a larger increase in CD8 density after nCRT (*p =* 0.006, paired Wilcoxon signed‐rank test) (Figure 6B).

Since biopsy and resection specimens are not entirely comparable due to heterogeneity within the tumor (Figure 6C), resection specimens of nCRT‐treated patients (*n* = 89) were compared with resections of patients treated by esophagectomy as a single treatment modality (*n* = 22). In nCRT‐treated patients, the mean CD8 density in the tumor epithelium compartment was not significantly increased compared with patients treated with surgery alone (732.8 versus 496.6, *p =* 0.16, Wilcoxon rank; supplementary material, Figure S4A). In contrast, the FOXP3 and PD‐1 mean densities were significantly lower in nCRT‐treated patients (*p =* 0.029 and *p* = 0.016) (supplementary material, Figure S4A).

Furthermore, TAIC densities in biopsy and resection specimens of patients treated with surgery alone were compared within the immune subtypes. Here, no significant increase in tumor epithelium located CD8^+^ TAICs was detected in the inflamed subtype (supplementary material, Figure S4B).

### Post‐treatment PD‐L1 expression of tumor and immune cells

Finally, the potential treatment effect on PD‐L1 expression on tumor cells and TAICs was assessed. In post‐nCRT resection specimens with remaining tumor, PD‐L1 expression was detected in 8.5% (*n* = 11/119) at low intensity, compared with 9.2% in pretreatment biopsies (supplementary material, Figure S5A). In patients with matched biopsies and post‐nCRT resections, 13.5% (*n* = 15/111) exhibited discordant PD‐L1 positivity, of which 6.3% (*n* = 7) were PD‐L1^+^ in the post‐treatment resection but negative in the biopsy (supplementary material, Figure S5E). PD‐L1^+^ TAICs were detected in an increased proportion of patients in post‐nCRT resections (58%, *n* = 69) compared with pretreatment biopsies and were largely located in the tumor epithelium (supplementary material, Figure S5B).

## Discussion

This is the first study to show that the tumor‐immune composition in pretreatment biopsies is associated with response to nCRT in EAC. This large well‐defined series of longitudinal collected matched pretreatment and post‐nCRT EAC samples provided a unique opportunity to explore variations in immune landscape patterns. Post‐nCRT, more intratumoral TAICs were seen, in particular more CD8^+^ TAICs. Furthermore, three distinct tumor‐immune landscape patterns could be identified in post‐nCRT resection specimens; the majority of tumors were inflamed.

### Post‐nCRT tumor‐immune landscape patterns

Applying a comprehensive image analysis of digital image whole slides, cell distribution patterns were conserved in the data. Significantly higher mean densities of CD8^+^ TAICs were detected in the tumor epithelium of inflamed tumors compared with pretreatment biopsies, further confirming former research demonstrating an influx of CD8^+^ immune cells after nCRT, or neoadjuvant chemotherapy alone, in EC [11, 29, 30, 31, 32]. This suggests that patients could benefit from the immune infiltration boost triggered by nCRT. This is of particular interest in view of recent immune‐directed treatment strategies such as PD‐1/PD‐L1 blockade [12, 13, 33, 34, 35, 36, 37, 38, 39, 40].

Moreover, 18% of tumors exhibited an invasive margin restricted phenotype. We hypothesize that the localization of TAICs at the tumor edge in patients with the invasive margin category does not sufficiently promote an active immune state [15]. Possibly, other immune‐suppressive cells, such as M2 macrophages or myeloid‐derived suppressor cells, barricade effector T‐cells at the edge of the tumor [41]. Indeed, a high abundance of CD68^+^ macrophages was detected in T‐cell‐excluded gastro‐EACs [42]. The extracellular matrix may additionally behave as a barrier [43]. Whether additional chemo(radiation) therapy could disrupt this biological border to enhance immune infiltration remains to be explored. Yet as we have demonstrated that nCRT according to the CROSS regimen does not sufficiently boost immune infiltration in this invasive margin restricted category, specific immune modulating strategies may be required to disrupt the restriction of TAICs to the edge of the tumor. In our study, PD‐L1 expression on tumor cells was not correlated to the spatial distribution patterns, suggesting the involvement of other immune‐suppressive pathways. Currently, immunotherapy directed at other immune checkpoints such as LAG‐3 and TIM‐3 is being evaluated in clinical trials and may also be of interest for EC [44].

We observed lower densities of FOXP3^+^ TAICs in the tumor compartment of nCRT‐treated patients compared with non‐nCRT‐treated resections. This has also been reported in a small set of EAC (*n* = 24) and ESC resection specimens, suggesting that an increased anti‐tumor‐immune landscape exists in the tumor center after chemoradiation [30]. Similar to our results, Zingg *et al* demonstrated that FOXP3 infiltration was not associated with outcome, suggesting that it might not influence patient survival to the extent previously hypothesized [45]. This is in line with the more dominant role for T‐cell exclusion rather than T‐cell suppression reported in chromosomal instable gastro‐EACs [42]. Likewise, in colorectal carcinoma, general T‐cell infiltration (CD3^+^ TAICs) in the tumor center and invasive margin was the best predictor for prognosis [46].

### PD‐L1 positivity

In our cohort, only a few EAC patients showed any PD‐L1 expression on tumor cells in pretreatment biopsies and resections post‐nCRT. Nonetheless, patients with a CPS ≥ 1 in pretreatment biopsies showed a better histopathological response to nCRT (TRG 1–3). CPS ≥ 10 has been shown to be a predictive marker for response to immunotherapy [40], but only four patients in our cohort remained positive after applying CPS ≥ 10, complicating further analysis. As predictive clinicopathological parameters and biomarkers are not well established for nCRT outcomes according to the CROSS regimen, further investigation of PD‐L1 as a biomarker for treatment response is desired. Timing after nCRT may be important, as PD‐L1 upregulation was observed to be transient after nCRT in preclinical studies [47]. This hampers data comparison, and may also explain the relatively low PD‐L1 positivity found in our cohort [48]. Yet it should be noted that the differences in PD‐L1 expression might also be attributed to intratumoral heterogeneity, which can only be partially captured in pretreatment biopsies. Possibly, the addition of TAIC cell density measurements, combined with other immune markers, could aid in the identification of patients eligible for neoadjuvant (immune) therapy.

### Strengths and limitations

Our study objectives were exploratory in nature; thus, a validation cohort was not included nor were multiple test corrections applied. The sample sizes in some of the immune subtypes were small, resulting in underpowered subgroup analysis. Nevertheless, until now, no cohort has been published of this sample size with digital image analysis of tumor sections in EAC with matched pretreatment biopsy and post‐treatment resection specimens. Even though the diagnostic biopsy sections were small in size and may not be entirely representative for the whole tumor due to intratumoral heterogeneity, they have been of value in our analysis. Moreover, predictive biomarkers will eventually have to be integrated in a clinical setting with diagnostics and treatment decisions before surgery, justifying the importance of exploring the immune cell patterns in biopsy specimens. Finally, the assessment of immune landscape patterns in relation to the tumor is not possible in resections with complete regression (TRG 1) because of the absence of vital tumor cells. The exclusion of complete responders could have introduced a bias and complicates extrapolation of the predictive value of CPS and CD8 density.

In conclusion, using a comprehensive digital whole slide image analysis, tumor cross‐section immune landscapes patterns were captured. Although assessment of the immune landscape is technically challenging in single biopsies of the primary tumor, high combined mean densities of CD8^+^, FOXP3^+^, and PD‐1^+^ TAICs in the tumor epithelium and tumor stroma compartments are associated with response to nCRT. This warrants future research into the potential of the tumor‐immune landscape for patient stratification and novel (immune) therapeutic strategies.

## Author contributions statement

AC, TS, BY, NG and HL were involved in the conception and design of the study. AC, TS, SM, MB, BY, NG and HL carried out the acquisition of data and/or analysis and interpretation of data. All the authors were involved in writing the paper and had final approval of the submitted and published versions.

## Supporting information


Supplementary materials and methods
**Figure S1.** Immune landscape in pretreatment EAC biopsies
**Figure S2.** PD‐L1^+^ EAC tumor cells in pretreatment biopsies
**Figure S3.** The difference in CD8^+^, FOXP3^+^, and PD‐1^+^ TAIC density in the tumor epithelium (T‐epithelium) and tumor stroma (T‐stroma) in pretreatment EAC biopsies between tumor regression grades (TRG) 1–3 and 4, 5
**Figure S4.** The difference in tumor‐immune landscape in resection specimens of nCRT‐treated patients versus those treated with surgery as a single treatment modality
**Figure S5.** PD‐L1^+^ EAC tumor cells in post‐nCRT resections
**Table S1.** Scoring system applied to assess TAIC density based on H&E stains using a 10× or 20× objective
**Table S2.** Scoring system applied to assess PD‐L1 expression on TAICs based on PD‐L1 stains using a 10× or 20× objective
**Table S3.** Threshold settings for detection of MHC I and MHC II using the Cytonuclear algorithm from Halo
**Table S4.** Cut‐off definitions for assignment of immune landscapes
**Table S5.** Mean density and ratio of TAICs in pretreatment biopsies and post‐treatment resection specimens
**Table S6.** Mean density and ratio of TAICs in pretreatment biopsies per TRG group
**Table S7.** Uni‐ and multi‐variate logistic regression model to predict TRG 1–3 versus TRG 4, 5 in pretreatment biopsies
**Table S8.** Uni‐ and multi‐variate Cox regression model to predict overall survival in pretreatment biopsies
**Table S9.** Uni‐ and multi‐variate Cox regression model to predict overall survival in resection specimens post‐nCRT
**Table S10.** The difference in mean density (cells/mm^2^) of CD8^+^, FOXP3^+^, and PD‐1^+^ TAICs and ratio of TAICs in tumor center and invasive margin per immune landscape pattern
**Table S11.** Color deconvolution vector values (referred to in Supplementary materials and methods)

## Data Availability

Data are available from the corresponding author on reasonable request.
