# Supplementary material for: Tumor‐immune landscape patterns before and after chemoradiation in resectable esophageal adenocarcinomas
Source: J Pathol. 2021 Dec 10;256(3):282–96. doi: 10.1002/path.5832 (PMC9299918; doi:10.1002/path.5832)
Supplement: Supplementary file 1 — Supplementary materials and methods Figure S1. Immune landscape in pretreatment EAC biopsies Figure S2. PD‐L1+ EAC tumor cells in pretreatment biopsies Figure S3. The difference in CD8+, FOXP3+, and PD‐1+ TAIC density in the tumor epithelium (T‐epithelium) and tumor stroma (T‐stroma) in pretreatment EAC biopsies between tumor regression grades (TRG) 1–3 and 4, 5 Figure S4. The difference in tumor‐immune landscape in resection specimens of nCRT‐treated patients versus those treated with surgery as a single treatment modality Figure S5. PD‐L1+ EAC tumor cells in post‐nCRT resections Table S1. Scoring system applied to assess TAIC density based on H&E stains using a 10× or 20× objective Table S2. Scoring system applied to assess PD‐L1 expression on TAICs based on PD‐L1 stains using a 10× or 20× objective Table S3. Threshold settings for detection of MHC I and MHC II using the Cytonuclear algorithm from Halo Table S4. Cut‐off definitions for assignment of immune landscapes Table S5. Mean density and ratio of TAICs in pretreatment biopsies and post‐treatment resection specimens Table S6. Mean density and ratio of TAICs in pretreatment biopsies per TRG group Table S7. Uni‐ and multi‐variate logistic regression model to predict TRG 1–3 versus TRG 4, 5 in pretreatment biopsies Table S8. Uni‐ and multi‐variate Cox regression model to predict overall survival in pretreatment biopsies Table S9. Uni‐ and multi‐variate Cox regression model to predict overall survival in resection specimens post‐nCRT Table S10. The difference in mean density (cells/mm2) of CD8+, FOXP3+, and PD‐1+ TAICs and ratio of TAICs in tumor center and invasive margin per immune landscape pattern Table S11. Color deconvolution vector values (referred to in Supplementary materials and methods) [file PATH-256-282-s001.docx]

**Tumor-immune landscape patterns before and after chemoradiation in resectable esophageal adenocarcinomas**

TTD Soeratram, A Creemers *et al. J Pathol* DOI: 10.1002/path.5832

**Supplementary materials and methods**

**Supplementary Figures S1–S5**

**Supplementary Tables S1–S11**

Reference numbers refer to the main text list

**Supplementary materials and methods**

**CD8 + FOXP3 + PD-1 + pan-cytokeratin multiplex immunohistochemistry**

Specimens were sectioned at 4-µm thickness, mounted onto positive-charged glass slides, dried, baked for 30 min at 60 °C, deparaffinized, and rehydrated. After a single antigen retrieval step, the CD8 + FOXP3 + PD-1 triplex IHC was sequentially performed with a heat-mediated antibody stripping procedure in between. All triplex IHC slides were scanned as digital images at an objective magnification of 20×. Coverslips of the multiplex IHC stained slides were removed by soaking in xylene for 4 days. Slides were then placed in 100% ethanol for an additional 3–4 days to remove the Vulcan Fast Red chromogen. The antigen retrieval and antibody stripping step removed the Vina Green chromogen. Epithelial/tumor cells were stained using the pan-cytokeratin antibody and subsequently digitized at 40× objective magnification.

**Validation of multiplex immunohistochemistry**

Both assays were designed and validated as a laboratory developed test by proprietary methods at Mosaic Laboratories (Lake Forest, CA, USA). The validation of the triplex assay was performed on FFPE tissues of tonsil (positive control), non-small cell lung cancer, breast cancer, and bladder cancer. The MHC I + MHC II duplex assay was validated on FFPE tissue of tonsil and esophageal cancer. The technical validity of both assays was achieved by staining serial sections of the multiplex assays in comparison to developing only one chromogen at a time, which allowed the check for cross-reactivity and the comparison of the results to expectations of the analytes individually. Images were captured from a 20× objective field of view in the same region across the slides and cells were scored digitally. The percentage of positive cells in the multiplex and single chromogen assays was comparable and cross-reactivity was not observed (data not shown). The reproducibility was verified by inter- and intra-day precision analyses. Images were captured from a 20× objective field of view in the same region across the slides and cells were scored digitally. The results of the inter and intra-day precision assays were comparable (data not shown).

**Quantification of PD-L1 staining**

Tumor cells were scored PD-L1 positive (i) if either partial or circumferential cell membrane staining was observed; cytoplasmic staining was not included. Staining intensity was scored as a percentage of PD-L1-expressing tumor cells on a scale ranging from 1+ to 3+.

Tumor-associated immune cells (TAICs) were scored for PD-L1 applying the same scoring system as that used for the tumor cells, selecting one to five representative regions of interest (ROIs) where lymphocytes, macrophages, and dendritic immune cells infiltrate the tumor, as clusters and/or as single cells in the tumor epithelium or tumor stroma. Specimens with PD-L1-positive immune cells were categorized according to the percentage of positive cells (supplementary material, Table S2).

**Quantification of the MHC I + MHC II duplex**

The MHC class I and II duplexes (ii) were scored by digital image analyses and visual quantification by a trained pathologist. For digital analyses, one ROI with tumor tissue intervening stroma and contiguous peritumoral stroma was selected, thus comprising both MHC class I and II cytoplasmic localization of tumor, immune, and positive stromal cells. Normal tissue, larger stromal areas, necrotic tissue, and staining artefacts were excluded from the analyses. The Cytonuclear IHC module from HALO image analysis software (Indica Labs, Albuquerque, NM, USA) was used to detect MHC expression in three different intensities (thresholds in supplementary material, Table S3). Data were categorized as the percentage of MHC I positive, MHC II positive, and dual positive (MHC I + MHC II) cells. The visual quantification was performed by a pathologist scoring the absence or presence of both membranous and cytoplasmic MHC I and MHC II class expression on tumor cells as either absent or present, including the percentage of cells.

**Whole slide image processing and analysis of CD8 + FOXP3 + PD-1 + pan-cytokeratin multiplex IHC**

The CD8 + FOXP3 + PD-1 multiplex slide was scanned using an Aperio ScanScope AT Turbo system (Aperio, Vista, CA, USA) with an objective of 20× and a resolution of 0.5025 μm/pixel. After de- and re-staining using the pan-cytokeratin antibody, the slides were scanned using a Philips IntelliSite Ultra-Fast Scanner (Philips Digital Pathology Solutions, Best, The Netherlands) with an objective of 40× and corresponding resolution of 0.25 μm/pixel. Whole slide images were loaded into QuPath v0.1.2 [22] for further processing. The tissue was detected with the tissue detection plugin of QuPath with varying settings. For each slide, the settings were optimized to obtain the best tissue detection. The tissue annotation was divided into annotation tiles of 2 mm^2^. The annotation tiles were transferred onto the pan-cytokeratin image after applying rotation (180°) and down sampling (2.01) to match the resolution of the scans.

*Tile selections in the ‘tumor center’ and ‘invasive margin’*

To recognize the spatial distribution of immune versus tumor cells, all tumor-containing tiles were selected manually in the whole slide images based on the pan-cytokeratin stain and histopathological morphology to exclude pan-cytokeratin-positive normal mucosa. Tumor-containing tiles selected in this way were manually classified as ‘tumor center’ or ‘invasive front’ tiles based on the following criteria. A ‘tumor center’ tile was defined as a tile that contained tumor cells covering the whole tile surface. All surrounding tiles also contained tumor. ‘Invasive margin’ tiles were located at the invasive margin or border of the tumor, which is the site where the tumor cells invade the normal tissue. Excluded sites were the normal mucosa and the dissection margins of the tissue specimen. ‘Invasive margin’ tiles contained tumor cells covering less than 50% of the tile surface. Adjacent tiles facing the outside of the border contained no tumor cells (non-tumor tiles). Adjacent tiles facing the inside of the border contained tumor cells (tumor tiles).

**Overlay of tumor annotation on multiplex staining**

*Alignment of pan-cytokeratin slide with multiplex slide*

All tiles that contained tumor (in invasive margin as well as tumor center) were manually selected together with the corresponding tile in the multiplex image and saved as an image stack. The image stacks were loaded in ImageJ2 [49] for the exact alignment of the tissue in the two different scans. To this end, the ‘Linear Stack Alignment with SIFT’ plugin for ImageJ2 was used, which is based on the Scale-Invariant Feature Transform (SIFT) algorithm developed by David Lowe [50]. The selected transformation setting was ‘Similarity’; this algorithm includes transformation by translation, rotation, and rescaling, but not shearing. The alignment tool created a corrected image of the pan-cytokeratin image where the multiplex image was used as the reference. The corrected pan-cytokeratin image was used for the detection and annotation of the tumor. A macro was written to automate this process.

*Color separation on tumor areas*

The red chromogen used to stain for pan-cytokeratin was separated from the background hematoxylin staining using the ‘Color deconvolution’ plugin developed by Landini *et al* [51]. The color separation used in the plugin is based on the algorithm published by Ruifrok and Johnston [52]. The default vector values for the separation of red chromogen from brown and blue were used (‘FastRed/FastBlue/DAB’). The output of the plugin was three separate 8-bit (grayscale) images for each chromogen.

*Positive pixel segmentation of tumor areas*

The 8-bit image for the red chromogen, the pan-cytokeratin staining, was smoothed with a Gaussian blur (sigma = 3) and median filter (radius = 2) to minimize background. Since the intensity of the staining varies across the slide, a variable threshold was chosen to segment the pixels into positive and negative pixels. The ‘MaxEntropy’ auto-threshold algorithm was selected as it had the best performance in varying conditions at different regions in different slides. After applying the threshold, a binary image mask was produced.

*Correction and detection of tumor areas*

As the pan-cytokeratin staining is cytoplasmic staining, nuclei are unstained and not included in the mask. To include ‘holes’ inside the tumor field annotation, the morphological filter ‘closing’ from the ‘MorphoLibJ’ plugin was applied (radius = 2) [53]. Holes larger than 80 µm^2^ were more likely to be a lumen inside a tumor gland and so were left out of the tumor annotation. The size of 80 µm^2^ was selected as the best performing cut-off after tests on multiple regions. The corrected binary mask was saved as ‘Region Of Interest’ (ROI) selection. In addition, the size in mm^2^ of the tumor areas and coordinates of the boundaries were saved.

*Positive pixel segmentation and detection of tumor nuclei*

The multiplex image was used to detect nuclei in the tumor, as the contrast between blue nuclei and blue background was better in this image. The nuclei were segmented with ‘Li’ auto-threshold, followed by median filter (radius = 2) and Gaussian blur filter (sigma = 1). In the binary image mask produced, ‘watershed’ was applied to separate attached cells that appeared as one ‘particle’. All particles that were inside the tumor ROI and exceeded the minimum size of 50 µm^2^ were counted as positive tumor nuclei in the plugin ‘Particle Analyzer’. The number of cells, coordinates (*XY* position in the whole slide) of the centroids, and average size were saved and exported to Excel.

*Color separation of CD8, FOXP3, and PD-1 chromogens*
Each 2-mm^2^ annotation tile from QuPath was opened using integrated ImageJ 1.49 [23] for further image processing. The following steps described were scripted in an ImageJ macro for automated analysis.
The chromogens used for the different cell types were separated with the color deconvolution plugin. The color deconvolution algorithm separates an image into three colors or ‘channels’. However, there are four colors on the multiplex image: Vina Green, Vulcan Red, DAB, and hematoxylin. As a consequence, the separated three ‘channels’ would contain contamination from the fourth color. We chose the vector values so that one color of interest was separated optimally, while the other two channels contained a mix of the remaining colors. We applied this strategy for each color of interest (Vina Green, Vulcan Red, and DAB). The optimal vector values to separate the colors were chosen based on tests on multiple regions in several slides (supplementary material, Table S11).

*Positive pixel segmentation of CD8, FOXP3, and PD-1*
After color deconvolution was applied, separate 8-bit images were produced for each cell type. As there is always a slight difference in staining intensity in whole resection slides, a flexible threshold was preferred over a static threshold. To segment the image into positive and negative pixel values, the automated threshold algorithm ‘Yen’ was used [54]. The optimal threshold algorithm was selected based on best performance in multiple regions that were distinct in intensities. The images were converted into binary images.
The separated DAB color image contained more background than the other chromogens. To reduce background, a Gaussian blur (sigma = 2) and median filter (radius = 2) were applied before setting the threshold for PD-1. Since PD-1-positive TAICs are less abundant than CD8-positive and FOXP3-positive TAICs, tiles can also be negative for PD-1. The automated threshold performed less accurately in this situation; hence, an upper limit threshold of 175 was used to prevent detecting background that would otherwise be ignored.

*Cell segmentation and detection of CD8, FOXP3, and PD-1*The watershed command was used to separate touching cells that were detected as one single particle. Afterwards the cell boundaries were smoothed with median filter (radius = 2). Positive ‘particles’ were then counted if they exceeded the minimum area of 10 µm^2^ to exclude background staining. A maximum size of 1000 µm^2^ was chosen to avoid excluding aggregates of cells that failed to separate with watershed. TAICs detected inside the previously saved tumor ROIs were labelled as cells in the tumor epithelium. TAICs outside the tumor ROIs were labelled as cells in the tumor stroma. If there was no tumor ROIs in the tile, the cells were labelled as cells in non-tumor stroma.

*Co-localization of CD8, FOXP3, and PD-1*Overlapping stains of different cell types were classified as double positive cells. This was achieved by creating an ROI for each cell type. For each cell type, the particle analysis was repeated inside the ROI of another cell type. If the particle was inside or at the border of the ROI, the cell was counted and labelled as double positive.

*Exported results*The coordinates of each detection (*X* and *Y* position in the whole slide) were saved, as well as the size (µm^2^) and perimeter. For each annotation tile, a summary file was created with the counts per cell type, average size (µm^2^), and label of the counts (tumor epithelium, tumor stroma or non-tumor stroma).

*Quality control*

The positive cell detections of each tile were projected as an annotation overlay in QuPath to visualize the cell detection in the whole slide. Each whole slide image was visually inspected to assess the performance of the ImageJ macro. Cases where the macro failed to detect clearly positively stained cells were excluded from further analyses.

**Analysis of cell densities per patient in R**

Data formatting and analysis were performed using R version 3.6.1 [55]. Data of each tile of a tumor were aggregated into one table. The R packages used to reshape the data in the appropriate format were ‘data.table’, ‘dplyr’, and ‘rlist’. The density of TAICs was determined by dividing the cell count by the tissue area of each tile. For TAICs in the tumor epithelium, the density was calculated by dividing the number of cells by the area of the tumor epithelium in each tile. For TAICs in the (non)tumor stroma, the number of cells was divided by the area of the stroma. The mean of all tile densities was calculated per patient. The ‘combined mean density’ was computed by taking the sum of the CD8, FOXP3, and PD-1 mean densities.

The CD8/(FOXP3 + PD-1) ratio was calculated by dividing the cell count of CD8 by the sum of the cell counts of FOXP3 and PD-1. The mean immune cell density was also calculated for the tumor center and for the invasive margin separately. The ratio of immune cells in the tumor center and invasive margin was calculated by dividing the mean density in the tumor center by the mean density in the invasive margin. Cut-offs were defined to separate the immune desert from the invasive margin and inflamed subtypes (supplementary material, Table S4).

Density heat map figures (Figure 5B–F) were created using the R package ‘spatstat’. Associations with survival data were performed with the R package ‘survival’. Boxplots and Kaplan–Meier figures were created with the R packages ‘ggplot2’ and ‘survminer’.

**
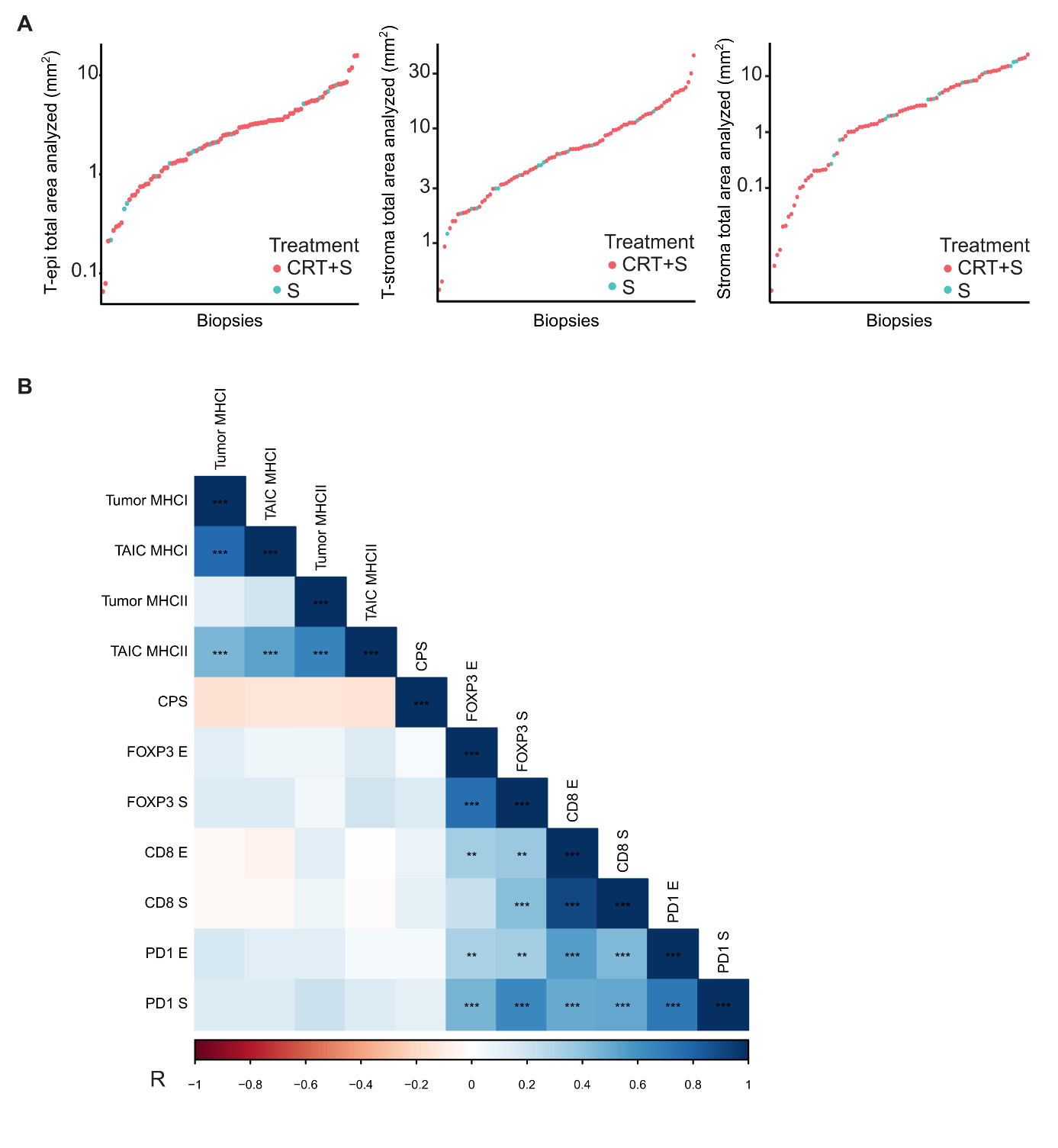
**

**Figure S1.** Immune landscape in pretreatment EAC biopsies. (A) The total analyzed area in mm^2^ in pretreatment biopsies in the tumor epithelium (T-epi), tumor stroma (T-stroma), and non-tumor stroma (Stroma). *Y*-axis in log_10_ scale. (B) Correlation plot of CD8^+^, FOXP3^+^, PD-1^+^, MHC I^+^, and MHC II^+^ TAICs; MHC I^+^ and MHC II^+^ tumor cells; and CPS in the tumor epithelium (E) and tumor stroma (S) compartment. The scale indicates the correlation coefficient *R*.

**
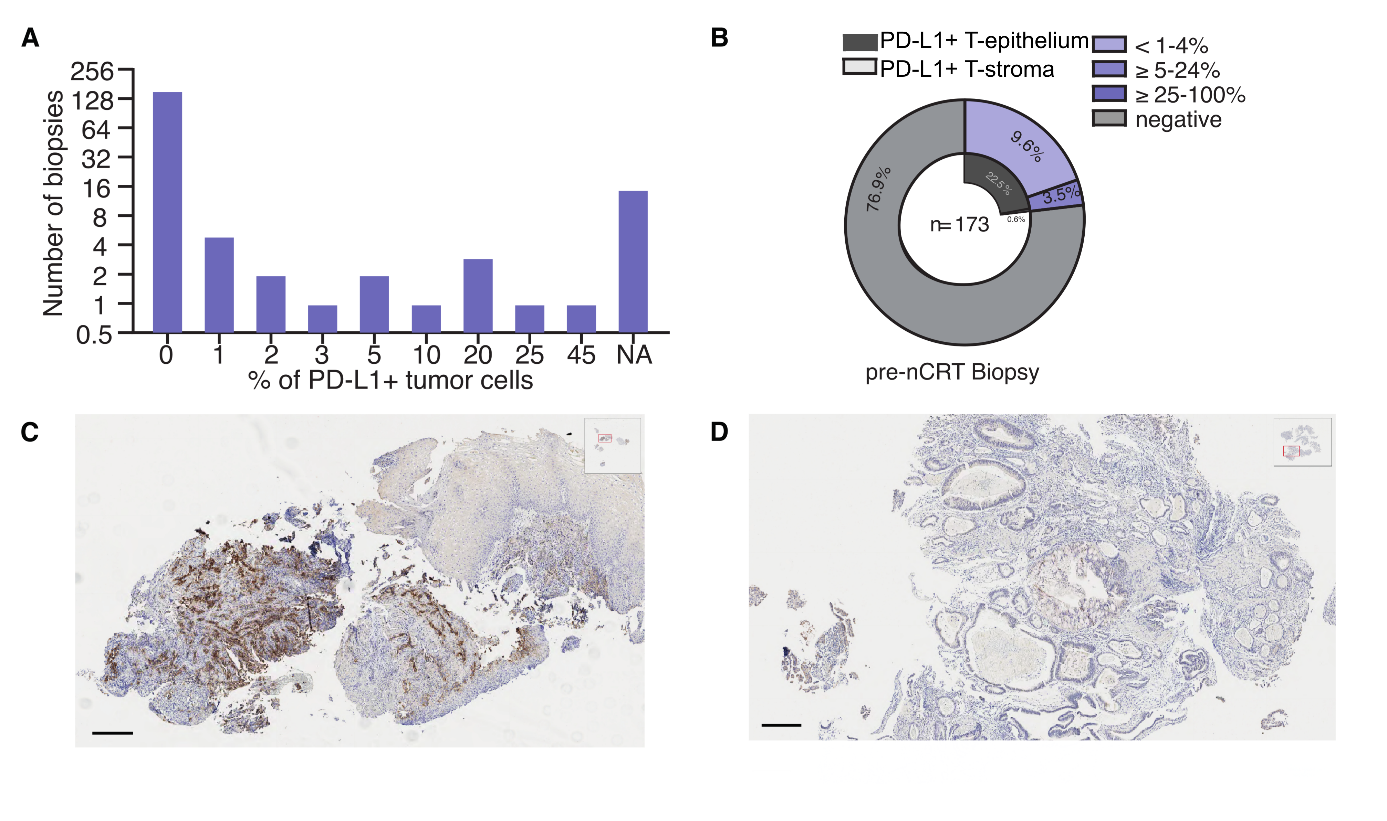
**

**Figure S2.** PD-L1^+^ EAC tumor cells in pretreatment biopsies. (A) The number of PD-L1-expressing pretreatment biopsies and the percentage of PD-L1-expressing tumor cells. (B) The percentage and location of pretreatment biopsies with PD-L1^+^ TAICs. Percentages in < 1–4%, ≥ 5–24%, ≥ 25-100%, or none of the TAICs. Location in tumor epithelium (T-epithelium) or tumor stroma (T-stroma). (C) Representative image of a pretreatment biopsy with PD-L1^+^ tumor cells. (D) Representative image of a PD-L1-negative pretreatment biopsy. Scale bars in C, D indicate 250 µm.

**
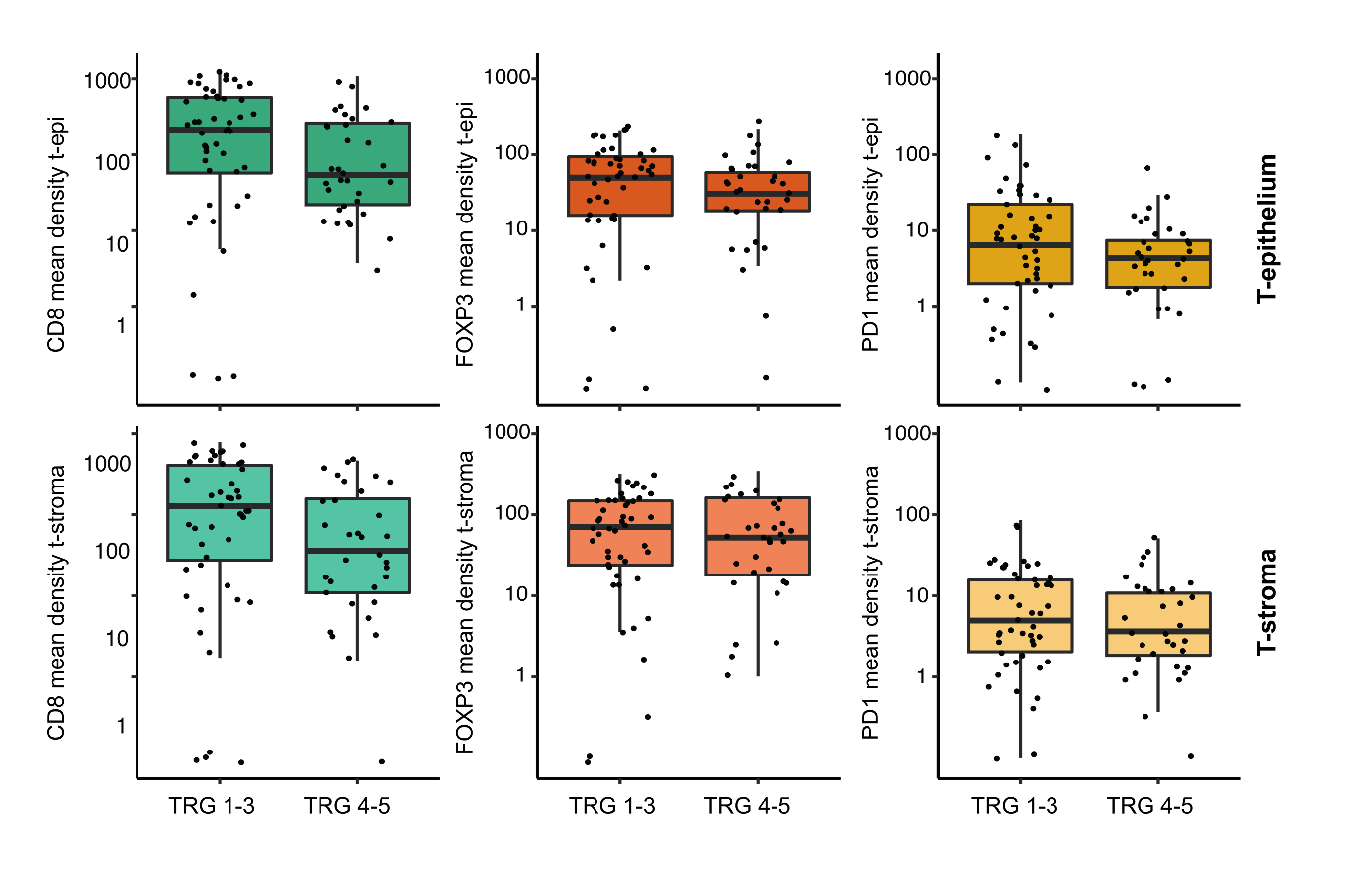
**

**Figure S3.** The difference in CD8^+^, FOXP3^+^, and PD-1^+^ TAIC density in the tumor epithelium (T-epithelium) and tumor stroma (T-stroma) in pretreatment EAC biopsies between tumor regression grades (TRG) 1–3 and 4, 5.

**
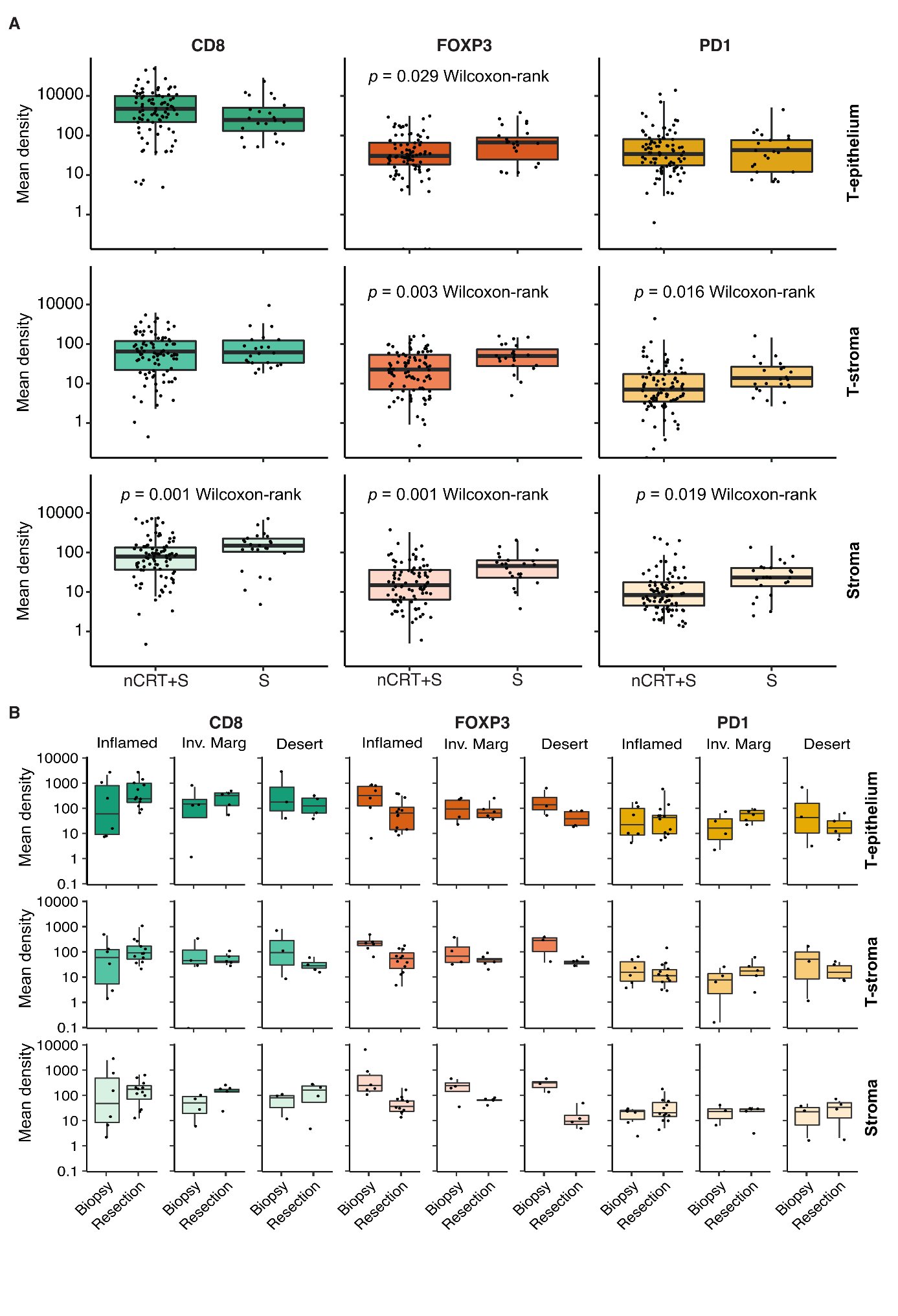
**

**Figure S4.** The difference in tumor-immune landscape in resection specimens of nCRT-treated patients versus those treated with surgery as a single treatment modality. (A) The mean difference in CD8^+^, FOXP3^+^, and PD-1^+^ TAIC density (cells/mm^2^) in the tumor epithelium, tumor stroma, and non-tumor stroma for EAC resections post-nCRT and treated with surgery (S) as a single treatment modality. A Wilcoxon rank-sum test was used to detect differences in mean TAIC density between nCRT- and S-treated patients. (B) The mean density of CD8^+^, FOXP3^+^, and PD-1^+^ TAICs (cells/mm^2^) in biopsies and resections of patients treated with surgery as a single treatment modality. TAIC densities are depicted per immune landscape pattern: inflamed, invasive margin (Inv. Marg), and desert, and separately for tumor epithelium (T-epithelium), tumor stroma (T-stroma), and non-tumor stroma (Stroma).

**
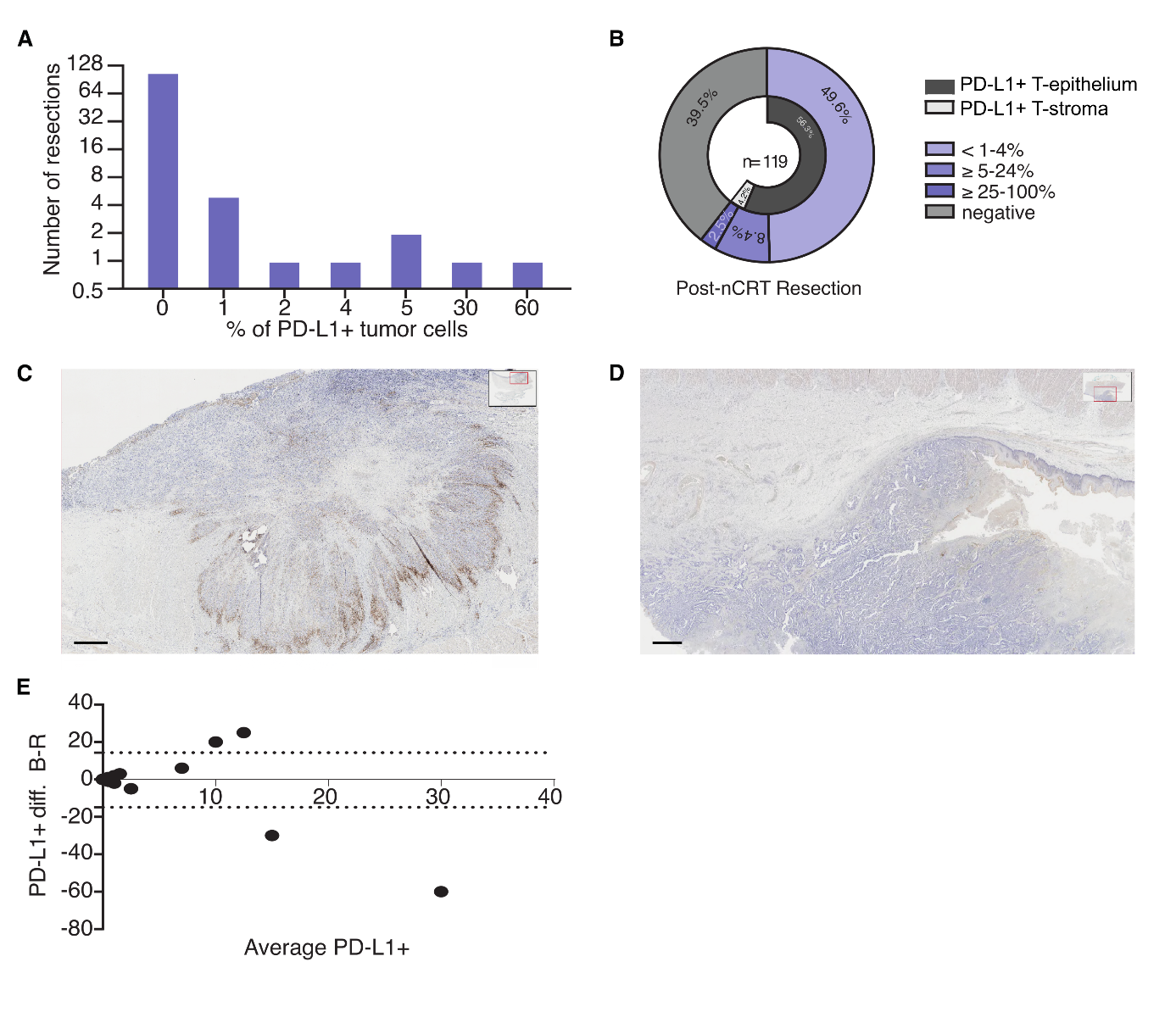
 Figure S5.** PD-L1^+^ EAC tumor cells in post-nCRT resections. (A) The number of PD-L1^+^ post-nCRT resections and the percentage of PD-L1^+^ tumor cells. (B) The percentage and location of post-treatment resections with PD-L1^+^ TAICs. Percentages in < 1–4%, ≥ 5–24%, ≥ 25–100%, or none of the TAICs. Location in tumor epithelium (T-epithelium) or tumor stroma (T-stroma). (C) Representative image of a post-nCRT resection with PD-L1^+^ tumor cells. Scale bar: 0.8 mm. (D) Representative image of a post-nCRT resection without PD-L1-expressing tumor cells. Scale bar: 1 mm. (E) A Blant–Altman plot of the difference versus the average PD-L1 positivity between pretreatment biopsies and post-nCRT resections. The dotted line indicates the 95% confidence interval (CI).

**Table S1.** Scoring system applied to assess TAIC density based on H&E stains using a 10× or 20× objective

| **H&E TAIC density** | **Small tissue specimens** | **Large tissue specimen** |
| --- | --- | --- |
| **Rare** | < 1% of the cells of the tumor compartment, 1 ROI selected | < 1% of the cells of the tumor compartment, 5 ROIs selected |
| **Intermediate** | 1–5% of the cells of the tumor compartment, 1 ROI selected | 1–5% of the cells of the tumor compartment, 5 ROIs selected |
| **Numerous** | > 5% of the cells of the tumor compartment, 1 ROI selected | > 5% of the cells of the tumor compartment, 5 ROIs selected |

**Table S2.** Scoring system to assess PD-L1 expression on TAICs based on PD-L1 stains using a 10× or 20× objective

| **PD-L1 TAIC density** | **Small tissue specimens** | **Large tissue specimen** |
| --- | --- | --- |
| **Rare** | < 1–4% of the cells of the tumor compartment, 1 ROI selected | < 1–4% of the cells of the tumor compartment, 5 ROIs selected |
| **Intermediate** | 5–24% of the cells of the tumor compartment, 1 ROI selected | 5–24% of the cells of the tumor compartment, 5 ROIs selected |
| **Numerous** | ≥ 25–100% of the cells of the tumor compartment, 1 ROI selected | ≥ 25–100% of the cells of the tumor compartment, 5 ROIs selected |

**Table S3.** Threshold settings for detection of MHC I and MHC II using the Cytonuclear algorithm from Halo

| **Intensity** | **MHC I** | **MHC II** |
| --- | --- | --- |
| **1+** | ≥ 0.05 – < 0.1 | ≥ 0.06 – < 0.15 |
| **2+** | ≥ 0.1 – < 0.15 | ≥ 0.15 – < 0.25 |
| **3+** | ≥ 0.15 | ≥ 0.25 |

**Table S4.** Cut-off definitions for assignment of immune landscapes

|  | **Ratio TC:IM < 0.5** | **Ratio TC:IM > 0.5** |
| --- | --- | --- |
| TC low (< 75 cells/mm^2^)  & IM low (< 400 intraepithelial cells/mm^2^ tumor) | Desert | Desert |
| TC low (< 75 cells/mm^2^)  & IM high (< 400 intraepithelial cells/mm^2^ tumor) | Invasive margin | Desert |
| TC intermediate (75–150 cells/mm^2^) | Invasive margin | Inflamed |
| TC high (> 150 cells/mm^2^) | Inflamed | Inflamed |

TC, tumor center; IM, inner margin.

**Table S5.** Mean density and ratio of TAICs in pretreatment biopsies and post-treatment resection specimens

|  | **Pretreatment biopsies *n* = 96** | | | | **Post-treatment resections *n* = 89** | | | | **Surgery only resections *n* = 22** | | | |
| --- | --- | --- | --- | --- | --- | --- | --- | --- | --- | --- | --- | --- |
|  | **Tumor epithelium** | **Tumor stroma** | **Stroma** | ***P* value** | **Tumor epithelium** | **Tumor stroma** | **Stroma** | ***P* value** | **Tumor epithelium** | **Tumor stroma** | **Stroma** | ***P* value** |
| **CD8 density** |  |  |  | **0.040** |  |  |  | **0.000** |  |  |  | **0.001** |
| Mean | 323.3 | 168.1 | 291.2 |  | 732.8 | 98.0 | 128.6 |  | 496.6 | 125.9 | 186.5 |  |
| Median | 158.8 | 85.5 | 91.0 |  | 438.5 | 64.4 | 79.4 |  | 246.0 | 61.2 | 149.5 |  |
| Min | 0.0 | 0.0 | 0.0 |  | 0.0 | 0.5 | 0.6 |  | 47.8 | 18.2 | 5.1 |  |
| Max | 2608.1 | 817.4 | 7662.8 |  | 5622.5 | 625.3 | 788.5 |  | 2903.9 | 948.8 | 679.6 |  |
| SD | 454.0 | 203.0 | 876.0 |  | 883.6 | 110.2 | 153.9 |  | 646.9 | 200.9 | 158.2 |  |
| **FOXP3 density** |  |  |  | 0.181 |  |  |  | **0.000** |  |  |  | 0.384 |
| Mean | 92.5 | 105.5 | 211.4 |  | 53.0 | 33.2 | 30.4 |  | 94.9 | 57.2 | 53.2 |  |
| Median | 49.2 | 68.9 | 50.3 |  | 30.4 | 22.7 | 14.9 |  | 66.4 | 50.0 | 45.5 |  |
| Min | 0.0 | 0.0 | 0.0 |  | 0.0 | 0.3 | 0.5 |  | 8.9 | 4.2 | 4.8 |  |
| Max | 888.5 | 457.6 | 6396.8 |  | 315.3 | 166.4 | 326.6 |  | 318.4 | 146.1 | 201.0 |  |
| SD | 146.6 | 102.4 | 769.1 |  | 60.8 | 34.0 | 44.4 |  | 95.4 | 39.6 | 43.5 |  |
| **PD-1 density** |  |  |  | **0.019** |  |  |  | **0.000** |  |  |  | **0.044** |
| Mean | 24.2 | 13.6 | 9.9 |  | 91.6 | 18.2 | 21.4 |  | 68.6 | 23.9 | 32.7 |  |
| Median | 6.0 | 5.0 | 3.0 |  | 33.5 | 6.8 | 8.4 |  | 42.6 | 13.8 | 23.2 |  |
| Min | 0.0 | 0.0 | 0.0 |  | 0.0 | 0.0 | 1.5 |  | 6.3 | 2.6 | 2.1 |  |
| Max | 582.5 | 191.0 | 115.9 |  | 1383.9 | 400.3 | 224.3 |  | 511.3 | 145.4 | 148.4 |  |
| SD | 66.7 | 24.2 | 17.0 |  | 201.5 | 45.5 | 40.9 |  | 107.6 | 30.5 | 32.3 |  |
| **Combined mean density** |  |  |  | 0.366 |  |  |  | **0.000** |  |  |  | **0.000** |
| Mean | 463.1 | 306.4 | 1148.3 |  | 882.9 | 152.7 | 181.4 |  | 664.8 | 208.0 | 276.9 |  |
| Median | 282.9 | 231.5 | 233.8 |  | 544.1 | 103.2 | 109.8 |  | 421.9 | 133.6 | 253.1 |  |
| Min | 0.0 | 0.6 | 0.0 |  | 0.0 | 7.4 | 5.4 |  | 80.6 | 55.2 | 17.3 |  |
| Max | 3326.4 | 1636.0 | 65527.9 |  | 7240.1 | 790.8 | 1044.3 |  | 3343.4 | 1130.6 | 768.9 |  |
| SD | 593.7 | 304.2 | 6890.5 |  | 1005.0 | 148.8 | 194.2 |  | 757.4 | 235.9 | 159.3 |  |
|  | **Pretreatment biopsies *n* = 93** | | | | **Post-treatment resections *n* = 87** | | | | **Surgery only resections *n* = 22** | | | |
| **Ratio CD8:(Foxp3 + PD-1)** | **Tumor epithelium** | **Tumor stroma** | ***P* value** | **Tumor total** | **Tumor epithelium** | **Tumor stroma** | ***P* value** | **Tumor total** | **Tumor epithelium** | **Tumor stroma** | ***P* value** | **Tumor total** |
|  |  |  | **0.003** |  |  |  | **0.009** |  |  |  | 0.203 |  |
| Mean | 13.0 | 13.2 |  | 10.7 | 13.2 | 7.1 |  | 8.8 | 7.1 | 3.6 |  | 4.7 |
| Median | 2.9 | 1.2 |  | 1.6 | 6.1 | 3.8 |  | 4.7 | 2.2 | 1.4 |  | 1.5 |
| Min | 0.0 | 0.0 |  | 0.0 | 0.0 | 0.0 |  | 0.0 | 0.2 | 0.3 |  | 0.3 |
| Max | 206.2 | 332.0 |  | 194.6 | 101.3 | 62.1 |  | 63.2 | 35.9 | 20.5 |  | 23.9 |
| SD | 31.7 | 44.2 |  | 32.0 | 17.0 | 9.6 |  | 11.6 | 9.5 | 5.0 |  | 6.3 |
|  | **Pretreatment biopsies *n* = 81 (nCRT only)** | | |  |  |  |  |  |  |  |  |  |
| **Low combined mean density (below median)** | **Tumor epithelium** | **Tumor stroma** |  |  |  |  |  |  |  |  |  |  |
| *N* | 40 | 40 |  |  |  |  |  |  |  |  |  |  |
| Mean | 92.2 | 75.0 |  |  |  |  |  |  |  |  |  |  |
| Median | 71.4 | 63.4 |  |  |  |  |  |  |  |  |  |  |
| Min | 0.0 | 0.6 |  |  |  |  |  |  |  |  |  |  |
| Max | 251.3 | 191.9 |  |  |  |  |  |  |  |  |  |  |
| SD | 70.2 | 53.9 |  |  |  |  |  |  |  |  |  |  |
| **High combined mean density (above median)** |  |  |  |  |  |  |  |  |  |  |  |  |
| *N* | 41 | 41 |  |  |  |  |  |  |  |  |  |  |
| Mean | 627.4 | 456.7 |  |  |  |  |  |  |  |  |  |  |
| Median | 480.0 | 394.1 |  |  |  |  |  |  |  |  |  |  |
| Min | 258.0 | 218.6 |  |  |  |  |  |  |  |  |  |  |
| Max | 1679.6 | 1063.1 |  |  |  |  |  |  |  |  |  |  |
| SD | 378.1 | 209.5 |  |  |  |  |  |  |  |  |  |  |

*P* values in bold are statistically significant.

**Table S6.** Mean density and ratio of TAICs in pretreatment biopsies per TRG group

|  | **Tumor epithelium** | | | | |  | **Tumor stroma** | | | | |  |
| --- | --- | --- | --- | --- | --- | --- | --- | --- | --- | --- | --- | --- |
|  | **TRG 2** | **TRG 3** | **TRG 4** | **TRG 5** | ***P* value ANOVA** | ***P* value chi-squared*** | **TRG 2** | **TRG 3** | **TRG 4** | **TRG 5** | ***P* value ANOVA** | ***P* value chi-squared*** |
|  | *n* = 11 | *n* = 37 | *n* = 28 | *n* = 5 |  |  | *n* = 11 | *n* = 37 | *n* = 28 | *n* = 5 |  |  |
| **CD8 density** | |  |  |  | 0.176 | **0.013** |  |  |  |  | 0.107 | **0.026** |
| Mean | 426.5 | 342.5 | 189.4 | 128.3 |  |  | 287.2 | 188.0 | 112.8 | 70.1 |  |  |
| Median | 488.8 | 199.0 | 58.7 | 33.1 |  |  | 341.8 | 116.4 | 41.1 | 14.2 |  |  |
| Min | 0.0 | 0.0 | 3.6 | 14.3 |  |  | 0.0 | 0.0 | 0.0 | 13.1 |  |  |
| Max | 909.3 | 1342.9 | 1083.1 | 336.8 |  |  | 626.0 | 787.0 | 471.3 | 243.7 |  |  |
| SD | 340.1 | 389.4 | 271.2 | 148.5 |  |  | 212.4 | 221.3 | 141.0 | 99.6 |  |  |
| **FOXP3 density** | |  |  |  | 0.434 | **0.048** |  |  |  |  | 0.189 | 0.238 |
| Mean | 76.8 | 61.2 | 59.5 | 21.9 |  |  | 105.7 | 88.2 | 90.9 | 51.0 |  |  |
| Median | 72.9 | 46.7 | 35.3 | 18.2 |  |  | 90.2 | 65.0 | 59.4 | 51.1 |  |  |
| Min | 3.4 | 0.0 | 0.7 | 0.0 |  |  | 25.7 | 0.0 | 0.9 | 2.1 |  |  |
| Max | 210.7 | 198.0 | 336.4 | 50.6 |  |  | 187.5 | 321.3 | 348.3 | 101.3 |  |  |
| SD | 57.9 | 57.4 | 73.8 | 21.3 |  |  | 55.6 | 89.1 | 89.3 | 44.4 |  |  |
| **PD-1 density** | |  |  |  | 0.541 | 0.222 |  |  |  |  | 0.237 | **0.048** |
| Mean | 15.6 | 21.0 | 8.7 | 4.9 |  |  | 9.5 | 12.9 | 10.1 | 2.0 |  |  |
| Median | 12.2 | 6.3 | 3.8 | 6.3 |  |  | 6.6 | 4.1 | 6.4 | 2.3 |  |  |
| Min | 0.2 | 0.0 | 0.0 | 0.9 |  |  | 2.0 | 0.0 | 0.0 | 1.2 |  |  |
| Max | 47.6 | 184.5 | 73.3 | 7.3 |  |  | 19.1 | 86.2 | 51.2 | 2.7 |  |  |
| SD | 16.0 | 39.1 | 14.5 | 2.7 |  |  | 6.5 | 19.3 | 12.1 | 0.6 |  |  |
| **Combined mean density** | | |  |  | **0.028** | **0.001** |  |  |  |  | 0.052 | **0.010** |
| Mean | 519.0 | 452.0 | 268.6 | 155.0 |  |  | 402.4 | 316.7 | 218.5 | 123.2 |  |  |
| Median | 543.6 | 259.9 | 98.0 | 58.8 |  |  | 469.9 | 219.4 | 146.2 | 79.5 |  |  |
| Min | 74.2 | 0.0 | 12.1 | 38.8 |  |  | 63.8 | 0.6 | 8.9 | 18.1 |  |  |
| Max | 1019.8 | 1679.6 | 1437.3 | 344.1 |  |  | 824.0 | 1636.0 | 740.6 | 347.2 |  |  |
| SD | 329.9 | 469.9 | 340.8 | 149.0 |  |  | 221.6 | 343.5 | 218.4 | 129.1 |  |  |
| **High combined mean density (above median)** | | | | | |  |  |  |  |  |  |  |
| *N* | 8 | 20 | 11 | 2 |  |  | 9 | 19 | 12 | 1 |  |  |
| **Low combined mean density (below median)** | | | | | |  |  |  |  |  |  |  |
| *N* | 3 | 17 | 17 | 3 |  |  | 2 | 18 | 16 | 4 |  |  |

*Linear-by-linear association chi-squared test. TAIC density was categorized into quartiles.

*P* values in bold are statistically significant.

**Table S7.** Uni- and multi-variate logistic regression model to predict TRG 1–3 versus TRG 4, 5 in pretreatment biopsies

|  | **Fisher’s exact test*** | | | **Univariate logistic regression** | | | | **Multivariate logistic regression** | | | |
| --- | --- | --- | --- | --- | --- | --- | --- | --- | --- | --- | --- |
|  | **Mandard low** | **Mandard high** | ***P* value** | **OR** | **95% CI lower** | **95% CI upper** | ***P* value** | **OR** | **95% CI lower** | **95% CI upper** | ***P* value** |
|  | ***n* = 48** | ***n* = 33** |  |  |  |  |  |  |  |  |  |
| **Age** |  |  | 0.498 | 0.967 | 0.919 | 1.019 | 0.209 | 1.042 | 0.975 | 1.113 | 0.229 |
| < 60 | 21 (65.6%) | 11 (34.4%) |  |  |  |  |  |  |  |  |  |
| > 60 | 27 (55.1%) | 22 (44.9%) |  |  |  |  |  |  |  |  |  |
| **T-stage** |  |  | 0.489 | 0.982 | 0.370 | 2.604 | 0.971 |  |  |  | 0.631 |
| 1 | 0 (0.0%) | 1 (100%) |  |  |  |  |  | 7.89E+08 | 0 | NA | 1 |
| 2 | 8 (72.7%) | 3 (27.3%) |  |  |  |  |  | 0.314 | 0.011 | 8.915 | 0.498 |
| 3 | 38 (57.6%) | 28 (42.4%) |  |  |  |  |  | 1.04 | 0.05 | 21.443 | 0.98 |
| 4 | 2 (66.7%) | 1 (33.3%) |  |  |  |  |  |  |  |  |  |
| **N-stage** |  |  | 0.980 | 0.945 | 0.525 | 1.700 | 0.850 |  |  |  | 0.83 |
| 0 | 11 (61.1%) | 7 (38.9%) |  |  |  |  |  | 0.973 | 0.076 | 12.431 | 0.984 |
| 1 | 33 (58.9%) | 23 (41.1%) |  |  |  |  |  | 0.664 | 0.061 | 7.19 | 0.736 |
| 3 | 4 (57.1%) | 3 (42.9%) |  |  |  |  |  |  |  |  |  |
| **CPS ≥ 1** |  |  | **0.010** | 0.157 | 0.033 | 0.745 | **0.020** | 0.094 | 0.013 | 0.676 | **0.019** |
| No | 34 (52.3%) | 31 (44.7%) |  |  |  |  |  |  |  |  |  |
| Yes | 14 (87.5%) | 2 (12.5%) |  |  |  |  |  |  |  |  |  |
| **CPS ≥ 10** |  |  | 0.142 | 0.000 | 0.000 | NA | 0.999 |  |  |  |  |
| No | 44 (57.1%) | 33 (42.9%) |  |  |  |  |  |  |  |  |  |
| Yes | 4 (100%) | 0 (0%) |  |  |  |  |  |  |  |  |  |
| **CD8 mean density tumor epithelium** | |  | **0.015** | 1.002 | 1.000 | 1.004 | **0.026** | 1 | 0.995 | 1.006 | 0.866 |
| Low | 16 (33.3%) | 20 (60.6%) |  |  |  |  |  |  |  |  |  |
| High | 32 (66.7%) | 13 (39.4%) |  |  |  |  |  |  |  |  |  |
| **FOXP3 mean density tumor epithelium** | |  | 0.225 | 1.003 | 0.995 | 1.011 | 0.439 | 0.999 | 0.983 | 1.016 | 0.943 |
| Low | 24 (50.0%) | 21 (63.6%) |  |  |  |  |  |  |  |  |  |
| High | 24 (50.0%) | 12 (36.4%) |  |  |  |  |  |  |  |  |  |
| **PD-1 mean density tumor epithelium** | |  | 0.191 | 1.026 | 0.994 | 1.059 | 0.111 | 0.971 | 0.909 | 1.037 | 0.382 |
| Low | 22 (45.8%) | 20 (60.6%) |  |  |  |  |  |  |  |  |  |
| High | 26 (54.2%) | 13 (39.4%) |  |  |  |  |  |  |  |  |  |
| **CD8 mean density tumor stroma** | |  | **0.049** | 1.003 | 1.000 | 1.006 | **0.025** | 0.994 | 0.986 | 1.003 | 0.221 |
| Low | 17 (35.4%) | 19 (57.6%) |  |  |  |  |  |  |  |  |  |
| High | 31 (64.6%) | 14 (42.4%) |  |  |  |  |  |  |  |  |  |
| **FOXP3 mean density tumor stroma** | |  | 0.261 | 1.001 | 0.996 | 1.007 | 0.693 | 1.003 | 0.99 | 1.017 | 0.627 |
| Low | 23 (47.9%) | 20 (60.6%) |  |  |  |  |  |  |  |  |  |
| High | 25 (52.1%) | 13 (39.4%) |  |  |  |  |  |  |  |  |  |
| **PD-1 mean density tumor stroma** | |  | 0.687 | 1.016 | 0.982 | 1.051 | 0.359 | 1.057 | 0.97 | 1.152 | 0.204 |
| Low | 24 (50.0%) | 18 (54.5%) |  |  |  |  |  |  |  |  |  |
| High | 24 (50.0%) | 15 (45.5%) |  |  |  |  |  |  |  |  |  |

*For Fisher’s exact test, CD8^+^, FOXP^+^ and PD-1^+^ TAIC densities were dichotomized according to the median value of the cohort.

Mean density in cells/mm^2^.

*P* values in bold are statistically significant.

**Table S8.** Uni- and multi-variate Cox regression model to predict overall survival in pretreatment biopsies

|  | **Fisher’s exact test** | | | **Univariate Cox regression** | | | | **Multivariate Cox regression** | | | |
| --- | --- | --- | --- | --- | --- | --- | --- | --- | --- | --- | --- |
|  | **Alive** | **Deceased** | ***P* value** | **HR** | **95% CI lower** | **95% CI upper** | ***P* value** | **HR** | **95% CI lower** | **95% CI upper** | ***P* value** |
|  | ***n* = 25** | ***n* = 56** |  |  |  |  |  |  |  |  |  |
| **Age** |  |  | 0.156 | 1.013 | 0.982 | 1.045 | 0.410 | 1.019 | 0.984 | 1.054 | 0.296 |
| < 60 | 3 (15.8%) | 15 (84.2%) |  |  |  |  |  |  |  |  |  |
| > 60 | 22 (35.5%) | 40 (64.5%) |  |  |  |  |  |  |  |  |  |
| **T-stage** |  |  | 0.090 |  |  |  | 0.356 |  |  |  | 0.421 |
| 1 | 0 (0.0%) | 1 (100%) |  | 3.260 | 0.202 | 52.570 | 0.405 | 3.464 | 0.187 | 64.316 | 0.405 |
| 2 | 6 (54.5%) | 5 (45.5%) |  | 1.307 | 0.152 | 11.208 | 0.807 | 1.955 | 0.200 | 19.150 | 0.565 |
| 3 | 17 (25.8%) | 49 (74.2%) |  | 2.686 | 0.370 | 19.497 | 0.328 | 3.623 | 0.438 | 29.979 | 0.232 |
| 4 (ref) | 2 (66.7%) | 1 (33.3%) |  |  |  |  |  |  |  |  |  |
| **N-stage** |  |  | 0.457 |  |  |  | 0.608 |  |  |  | 0.999 |
| 0 | 7 (38.9%) | 11 (61.1%) |  | 0.946 | 0.301 | 2.975 | 0.925 | 0.974 | 0.242 | 3.922 | 0.971 |
| 1 | 15 (26.8%) | 41 (73.2%) |  | 1.297 | 0.464 | 3.626 | 0.620 | 0.970 | 0.274 | 3.439 | 0.963 |
| 3 (ref) | 3 (42.9%) | 4 (57.1%) |  |  |  |  |  |  |  |  |  |
| **CPS ≥ 1** |  |  | 0.077 | 1.889 | 0.893 | 3.999 | 0.096 | 2.210 | 0.848 | 5.761 | 0.105 |
| No | 17 (26.2%) | 48 (73.8%) |  |  |  |  |  |  |  |  |  |
| Yes (ref) | 8 (50%) | 8 (50%) |  |  |  |  |  |  |  |  |  |
| **CPS ≥ 10** |  |  | 1.000 | 0.823 | 0.257 | 2.637 | 0.742 |  |  |  |  |
| No | 24 (31.2%) | 53 (68.8%) |  |  |  |  |  |  |  |  |  |
| Yes | 1 (25%) | 3 (75%) |  |  |  |  |  |  |  |  |  |
| **CD8 mean density tumor epithelium** | | | 0.591 | 1.000 | 0.999 | 1.001 | 0.940 | 1.003 | 1.000 | 1.005 | **0.020** |
| Low | 10 (27.8%) | 26 (72.2%) |  |  |  |  |  |  |  |  |  |
| High | 15 (33.3%) | 30 (66.7%) |  |  |  |  |  |  |  |  |  |
| **FOXP3 mean density tumor epithelium** | | | 0.361 | 1.000 | 0.996 | 1.005 | 0.889 | 1.003 | 0.995 | 1.011 | 0.464 |
| Low | 12 (26.7%) | 33 (73.3%) |  |  |  |  |  |  |  |  |  |
| High | 13 (36.1%) | 23 (63.9%) |  |  |  |  |  |  |  |  |  |
| **PD-1 mean density tumor epithelium** | | | 0.144 | 0.999 | 0.989 | 1.008 | 0.783 | 0.997 | 0.975 | 1.020 | 0.799 |
| Low | 16 (38.1%) | 26 (61.9%) |  |  |  |  |  |  |  |  |  |
| High | 9 (23.1%) | 30 (76.9%) |  |  |  |  |  |  |  |  |  |
| **CD8 mean density tumor stroma** | |  | 0.591 | 0.999 | 0.998 | 1.001 | 0.352 | 0.996 | 0.992 | 1.000 | **0.039** |
| Low | 30 (53.6%) | 26 (46.6%) |  |  |  |  |  |  |  |  |  |
| High | 15 (60%) | 10 (40%) |  |  |  |  |  |  |  |  |  |
| **FOXP3 mean density tumor stroma** | | | 0.115 | 0.998 | 0.995 | 1.002 | 0.272 | 0.999 | 0.992 | 1.007 | 0.859 |
| Low | 33 (58.9%) | 23 (41.1%) |  |  |  |  |  |  |  |  |  |
| High | 10 (40%) | 15 (60%) |  |  |  |  |  |  |  |  |  |
| **PD-1 mean density tumor stroma** | |  | 0.643 | 0.987 | 0.966 | 1.008 | 0.231 | 0.992 | 0.950 | 1.036 | 0.721 |
| Low | 26 (46.4%) | 30 (53.6%) |  |  |  |  |  |  |  |  |  |
| High | 13 (52%) | 12 (48%) |  |  |  |  |  |  |  |  |  |

*For Fisher’s exact test, CD8^+^, FOXP^+^, and PD-1^+^ TAIC densities were dichotomized according to the median value of the cohort.
Mean density in cells/mm^2^.

*P* values in bold are statistically significant.

**Table S9.** Uni- and multi-variate Cox regression model to predict overall survival in resection specimens post-nCRT

|  | **Pearson’s chi-squared** | | | **Univariate Cox regression** | | | | **Multivariate Cox regression** | | | |
| --- | --- | --- | --- | --- | --- | --- | --- | --- | --- | --- | --- |
|  | **Alive** | **Deceased** | ***P* value** | **HR** | **95% CI lower** | **95% CI upper** | ***P* value** | **HR** | **95% CI lower** | **95% CI upper** | ***P* value** |
|  | ***n* = 24** | ***n* = 66** |  |  |  |  |  | 1.005 | 0.975 | 1.036 | 0.740 |
| **Age** |  |  | 0.512 | 1.001 | 0.972 | 1.030 | 0.965 |  |  |  |  |
| < 60 | 19 (28.8%) | 47 (71.2%) |  |  |  |  |  |  |  |  |  |
| > 60 | 5 (21.7%) | 18 (78.3%) |  |  |  |  |  |  |  |  |  |
| **T-stage** |  |  | 0.143 |  |  |  | 0.316 |  |  |  | 0.575 |
| 1 |  |  |  |  |  |  |  | 0.442 | 0.079 | 2.484 | 0.354 |
| 2 | 6 (50.0%) | 6 (50.0%) |  | 0.437 | 0.088 | 2.172 | 0.311 | 0.660 | 0.143 | 3.042 | 0.594 |
| 3 | 17 (23.0%) | 57 (77.0%) |  | 0.824 | 0.201 | 3.387 | 0.789 |  |  |  |  |
| 4 (reference) | 1 (33.3%) | 2 (66.7%) |  |  |  |  |  |  |  |  |  |
| **N-stage** |  |  | 0.518 |  |  |  | 0.523 |  |  |  | 0.285 |
| 0 | 8 (36.5%) | 14 (63.5%) |  | 0.691 | 0.265 | 1.803 | 0.450 | 0.631 | 0.214 | 1.858 | 0.404 |
| 1 | 14 (23.7%) | 45 (76,3%) |  | 0.971 | 0.413 | 2.281 | 0.946 | 1.064 | 0.413 | 2.744 | 0.897 |
| 3 (reference) | 2 (25.0%) | 6 (75.0%) |  |  |  |  |  |  |  |  |  |
| **TRG** |  |  | 0.934 | 0.927 | 0.562 | 1.529 | 0.767 | 0.926 | 0.546 | 1.572 | 0.776 |
| 1–3 | 15 (27.3%) | 40 (72.7%) |  |  |  |  |  |  |  |  |  |
| 4, 5 (reference) | 9 (26.5%) | 25 (73.5%) |  |  |  |  |  |  |  |  |  |
| **Immune landscape** |  |  | 0.253 |  |  |  | 0.215 |  |  |  | 0.181 |
| Inflamed | 17 (34.7%) | 32 (65.3%) |  | 0.605 | 0.337 | 1.087 | 0.093 | 0.561 | 0.300 | 1.051 | 0.071 |
| Invasive margin | 3 (18.8%) | 13 (81.3%) |  | 0.845 | 0.411 | 1.734 | 0.645 | 0.825 | 0.377 | 1.807 | 0.631 |
| Desert (reference) | 4 (18.2%) | 18 (81.8%) |  |  |  |  |  |  |  |  |  |
| **Total mean density tumor epithelium in tumor center** | | |  | 1.000 | 0.999 | 1.000 | 0.163 |  |  |  |  |
| **Total mean density tumor stroma in tumor center** | | |  | 1.000 | 0.998 | 1.001 | 0.620 |  |  |  |  |
| **CD8 mean density tumor epithelium in tumor center** | | |  | 1.000 | 0.999 | 1.000 | 0.184 |  |  |  |  |
| **FOXP3 mean density tumor epithelium in tumor center** | | |  | 1.005 | 0.989 | 1.002 | 0.151 |  |  |  |  |
| **PD-1 mean density tumor epithelium in tumor center** | | |  | 1.002 | 0.995 | 1.001 | 0.228 |  |  |  |  |

**Table S10.** The difference in mean density (cells/mm^2^) of CD8^+^, FOXP3^+^, and PD-1^+^ TAICs and ratio of TAICs in tumor center and invasive margin per immune landscape pattern

|  | **Tumor epithelium** | | | | **Tumor stroma** | | | | **Tumor total** | | | |
| --- | --- | --- | --- | --- | --- | --- | --- | --- | --- | --- | --- | --- |
|  | **Inflamed** | **Invasive margin** | **Desert** | ***P* value** | **Inflamed** | **Invasive margin** | **Desert** | ***P* value** | **Inflamed** | **Invasive margin** | **Desert** | ***P* value** |
|  | ***n* = 49** | ***n* = 16** | ***n* = 22** |  | ***n* = 49** | ***n* = 16** | ***n* = 22** |  | ***n* = 49** | ***n* = 16** | ***n* = 22** |  |
| **CD8** |  |  |  | **0.000** |  |  |  | **0.000** |  |  |  | **0.000** |
| Mean | 1022.1 | 461.0 | 333.5 |  | 136.6 | 49.5 | 27.5 |  | 1158.7 | 510.5 | 361.0 |  |
| Median | 706.4 | 347.5 | 140.4 |  | 95.5 | 50.8 | 20.8 |  | 833.5 | 429.7 | 173.9 |  |
| Min | 0.0 | 58.6 | 5.6 |  | 6.4 | 3.3 | 0.9 |  | 6.4 | 111.8 | 6.5 |  |
| Max | 5622.5 | 1352.6 | 1593.7 |  | 414.3 | 118.0 | 76.7 |  | 5825.0 | 1400.3 | 1646.0 |  |
| SD | 1051.0 | 351.3 | 458.6 |  | 105.2 | 36.6 | 22.6 |  | 1087.4 | 367.3 | 473.0 |  |
| **FOXP3** |  |  |  | 0.103 |  |  |  | 0.057 |  |  |  | 0.101 |
| Mean | 56.5 | 65.0 | 27.3 |  | 38.2 | 19.3 | 29.4 |  | 94.7 | 84.2 | 56.7 |  |
| Median | 32.4 | 27.6 | 25.9 |  | 28.0 | 14.2 | 12.5 |  | 58.5 | 45.0 | 41.1 |  |
| Min | 0.0 | 5.2 | 0.0 |  | 0.3 | 2.5 | 3.5 |  | 0.3 | 8.4 | 7.6 |  |
| Max | 233.7 | 315.3 | 80.8 |  | 163.8 | 63.9 | 166.4 |  | 296.5 | 325.7 | 166.4 |  |
| SD | 54.1 | 84.6 | 19.0 |  | 34.3 | 19.2 | 39.7 |  | 75.3 | 91.9 | 44.1 |  |
| **PD-1** |  |  |  | 0.756 |  |  |  | 0.059 |  |  |  | 0.561 |
| Mean | 124.6 | 44.1 | 52.1 |  | 25.3 | 7.3 | 9.1 |  | 149.9 | 51.4 | 61.2 |  |
| Median | 43.5 | 29.5 | 32.4 |  | 10.0 | 4.5 | 5.6 |  | 50.4 | 35.5 | 42.8 |  |
| Min | 0.0 | 3.0 | 0.0 |  | 0.0 | 0.5 | 0.3 |  | 0.0 | 3.8 | 4.3 |  |
| Max | 1383.9 | 160.9 | 283.7 |  | 400.3 | 21.2 | 53.5 |  | 1449.2 | 165.5 | 294.9 |  |
| SD | 263.5 | 42.3 | 61.8 |  | 59.6 | 6.7 | 11.6 |  | 291.8 | 44.3 | 61.2 |  |
| **Ratio TC:IM** | |  |  | **0.000** |  |  |  | 0.055 |  |  |  | **0.001** |
| Mean | 1.7 | 0.2 | 1.1 |  | 43.6 | 13.1 | 12.2 |  | 8.8 | 1.8 | 3.8 |  |
| Median | 0.8 | 0.2 | 1.0 |  | 7.2 | 1.8 | 3.1 |  | 4.7 | 1.8 | 1.5 |  |
| Min | 0.1 | 0.1 | 0.0 |  | 0.1 | 0.0 | 0.0 |  | 0.5 | 0.0 | 0.1 |  |
| Max | 15.1 | 0.5 | 2.8 |  | 1086.6 | 171.8 | 76.2 |  | 54.8 | 4.5 | 25.7 |  |
| SD | 2.9 | 0.1 | 0.7 |  | 171.1 | 42.4 | 22.3 |  | 11.3 | 1.3 | 6.0 |  |
| **Combined mean density** | | |  | **0.000** |  |  |  | **0.000** |  |  |  | **0.000** |
| Mean | 1203.2 | 570.1 | 412.8 |  | 200.1 | 76.0 | 66.0 |  | 1403.4 | 646.1 | 478.8 |  |
| Median | 930.5 | 463.4 | 197.8 |  | 157.0 | 73.6 | 45.2 |  | 1172.0 | 550.6 | 293.2 |  |
| Min | 0.0 | 155.6 | 35.8 |  | 7.4 | 7.6 | 7.6 |  | 7.4 | 163.3 | 74.9 |  |
| Max | 7240.1 | 1365.8 | 1714.2 |  | 527.2 | 159.0 | 258.0 |  | 7561.1 | 1417.5 | 1805.6 |  |
| SD | 1213.2 | 316.1 | 494.2 |  | 132.4 | 41.4 | 58.9 |  | 1245.8 | 323.0 | 502.5 |  |
| **Ratio CD8:(Foxp3 + PD-1)** | | |  | **0.002** |  |  |  | **0.022** |  |  |  | **0.009** |
| Mean | 17.0 | 12.9 | 5.1 |  | 8.5 | 7.0 | 3.0 |  | 10.9 | 8.8 | 3.5 |  |
| Median | 10.5 | 4.8 | 2.9 |  | 4.7 | 5.4 | 1.9 |  | 5.9 | 5.1 | 2.3 |  |
| Min | 0.3 | 1.1 | 0.2 |  | 0.2 | 0.6 | 0.1 |  | 0.2 | 0.8 | 0.1 |  |
| Max | 101.3 | 48.8 | 26.0 |  | 62.1 | 19.6 | 10.0 |  | 63.2 | 28.4 | 12.1 |  |
| SD | 19.6 | 15.4 | 6.4 |  | 11.4 | 6.1 | 2.8 |  | 13.8 | 8.9 | 3.5 |  |

TC, tumor center; IM, invasive margin.

*P* values in bold are statistically significant.

**Table S11.** Color deconvolution vector values

|  | R1 | G1 | B1 | R2 | G2 | B2 | R3 | G3 | B3 |
| --- | --- | --- | --- | --- | --- | --- | --- | --- | --- |
| CD8/ Vina Green | 0.80899334 | 0.4471633 | 0.38154262 | 0.6250343 | 0.6430759 | 0.4424767 | 0.4519506 | 0.56458837 | 0.6906378 |
| FOXP3/ Vulcan Red | 0.34260333 | 0.73534805 | 0.58471036 | 0.6653873 | 0.62918997 | 0.40172097 | 0.44462672 | 0.5611227 | 0.6981751 |
| PD-1/ DAB | 0.98051363 | 0.14438722 | 0.1332117 | 0.26814753 | 0.57031375 | 0.77642715 | 0.001 | 0.79549694 | 0.60595757 |
